# Cooperative interactions among females can lead to even more extraordinary sex ratios

**DOI:** 10.1002/evl3.217

**Published:** 2021-06-03

**Authors:** Ryosuke Iritani, Stuart A. West, Jun Abe

**Affiliations:** ^1^ Interdisciplinary Theoretical and Mathematical Sciences (iTHEMS) RIKEN Wako 351‐0198 Japan; ^2^ Department of Zoology University of Oxford Oxford OX1 3PS United Kingdom; ^3^ Faculty of Liberal Arts Meiji Gakuin University Yokohama 244–8539 Japan

**Keywords:** Cooperation, Kin selection, Local resource competition, Local mate competition, Local resource enhancement, Sex‐biased dispersal

## Abstract

Hamilton's local mate competition theory provided an explanation for extraordinary female‐biased sex ratios in a range of organisms. When mating takes place locally, in structured populations, a female‐biased sex ratio is favored to reduce competition between related males, and to provide more mates for males. However, there are a number of wasp species in which the sex ratios appear to more female biased than predicted by Hamilton's theory. It has been hypothesized that the additional female bias in these wasp species results from cooperative interactions between females. We investigated theoretically the extent to which cooperation between related females can interact with local mate competition to favor even more female‐biased sex ratios. We found that (i) cooperation between females can lead to sex ratios that are more female biased than predicted by local competition theory alone, and (ii) sex ratios can be more female biased when the cooperation occurs from offspring to mothers before dispersal, rather than cooperation between siblings after dispersal. Our models formally confirm the verbal predictions made in previous experimental studies, which could be applied to a range of organisms. Specifically, cooperation can help explain sex ratio biases in *Sclerodermus* and *Melittobia* wasps, although quantitative comparisons between predictions and data suggest that some additional factors may be operating.

**Impact Summary**

In many animals, it is well established that natural selection stabilizes the production of equal sex ratios. In some insects, the sex ratios may be significantly biased to females. William D Hamilton's theory provides an explanation for female‐biased sex ratios: if sons’ dispersal capacity is limited, they may end up with competing for mating opportunity, which is disadvantageous because they may be brothers sharing the same genes inherited from mother. This process, coined “local mate competition,” is known to result in extremely female‐biased sex ratios, with the well‐known formula for the sex ratio of *x* = (*n* − 1)∕(2*n*) < 1∕2 (for diploidy), in which a decrease in the number of females *n* ovipositing in the same patch results in lower sex ratios. Yet, even more female‐biased sex ratios are observed in *Sclerodermus harmandi* and *Melittobia australica* wasp species, where females have been suggested to engage in cooperative behaviors when attacking their host species. This study carries out mathematical analyses and challenges this puzzle by incorporating such female‐female communal interactions, termed “local resource enhancement” (LRE). We found that LRE can, as expected, lead to even more female‐biased sex ratios from Hamilton's predictions. Although a quantitative discrepancy from the data in these species remains large, our predictions help elucidate how LRE can favor female‐biased sex ratios, as well as provide modeling framework to incorporate various kinds of social interactions across sexes.

Sex ratio theory has provided one of the most productive and successful areas of evolutionary biology (Charnov 1982; Hardy 2002; West 2009). Theory predicts a number of situations in which individuals are expected to adjust the sex ratios of their offspring in response to local conditions (Charnov 1982; Frank 1998). This theory has been applied to explain variation in the offspring sex ratio (proportion males) across a range of taxa, from malaria parasites to ants to birds (Bourke and Franks 1995; Hardy 2002; West 2009).

One of the major challenges is to explain when sex ratios are biased away from equal investment in the sexes. Hamilton's (1967) local mate competition (LMC) theory provides a conceptual explanation for female‐biased sex ratios observed in parasitic wasps (e.g., *Scelionidae*, *Alfonsiella*, *Apanteles*, and *Nasonia*), aphids (e.g., *Prociphilus oriens*), and a number of fig wasps (Waage 1982; Greeff 2002; Tagawa 2000; Gu and Dorn 2003; Werren 1983; Shuker et al. 2006; Burton‐Chellew et al. 2008; Yamaguchi 1985; Herre 1985). Specifically, Hamilton showed that if (i) *n* diploid females lay eggs in a patch, (ii) males do not disperse, and (iii) mating occurs before all females disperse, then the evolutionarily stable strategy (ESS; Maynard Smith and Price 1973) is to produce an offspring sex ratio of (*n* − 1)/(2*n*) (Fig. 1), which predicts female‐biased offspring sex ratios (smaller than 1/2) and becomes less biased as more females lay eggs in a patch (i.e., as *n* increases). Succeeding work (Taylor 1981, 1988, 1992; Bulmer 1986; Frank 1986b; 1998) made it clear that in Hamilton's LMC theory, selection on the female bias is mediated by the balance among three factors: (i) a benefit for reduced competition between sons, (ii) a benefit for production of more mates (daughters) for those sons (“mating bonus”; Frank 1998), and (iii) a cost for stronger local resource competition among females (LRC; Clark 1978). Hamilton's LMC theory has been extremely successful in explaining variation in the offspring sex ratio, both across species and between individuals (Taylor 1981, 1994; Avilés 1993; Gardner and West 2006; Shuker et al. 2004, 2006; Gardner et al. 2009; West 2009; Rodrigues and Gardner 2015; Gardner and Hardy 2021).

**Figure 1:**
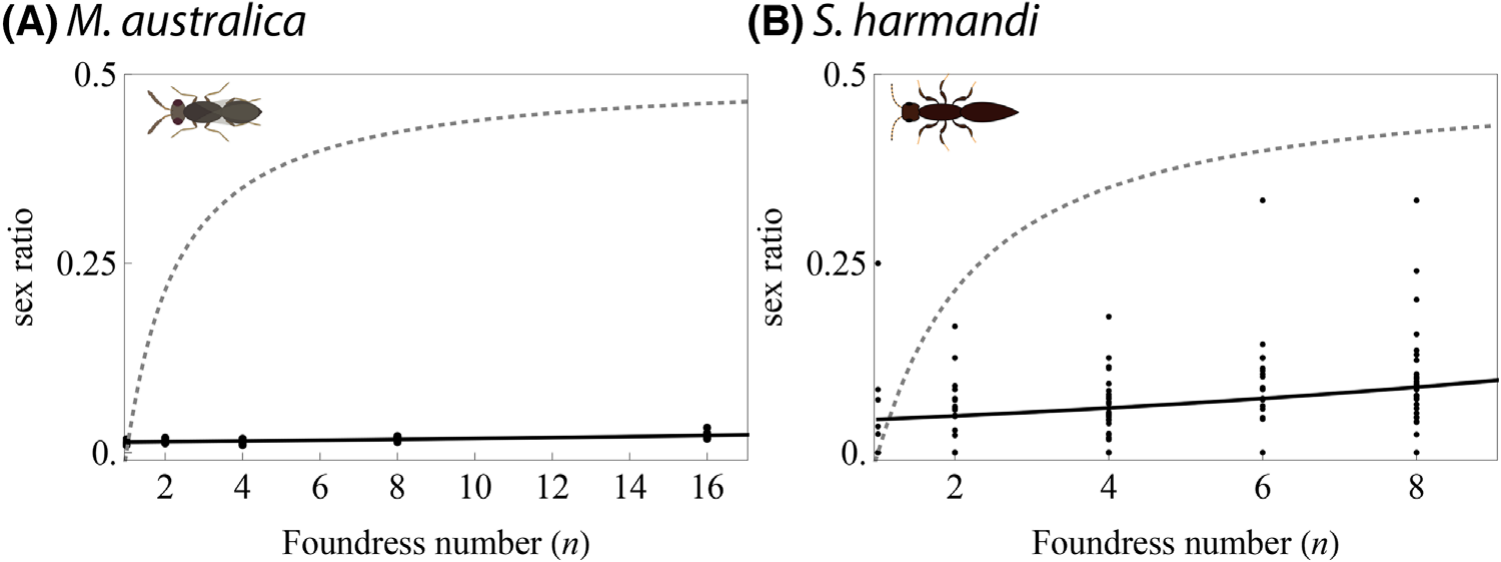
Extremely female‐biased sex ratio in (A) *Melittobia australica* from Abe et al. (2003), and (B) *Sclerodermus harmandi* from Tang et al. (2014) and Kapranas et al. (2016). Both species are haplodiploids. Outliers removed for (B), as in the original articles Tang et al. (2014) and Kapranas et al. (2016). Note that the horizontal axes are scaled differently. Dotted lines: Reference sex ratio given by (*n* − 1)(4*n* − 2)/(2*n*(4*n* − 1)) (evolutionarily stable sex ratio for haplodiploids with *d*_f_ = 1). Solid line in panel A: predicted values by generalized linear models; in panel B: shown in Tang et al. (2014). For *M. australica* in a natural population, the foundress number varied from 1 to 36 (with mean 6.7 and standard deviation 10.0).

However, there are a number of cases where females produce extremely female‐biased offspring sex ratios, which do not appear to be completely explained by LMC theory alone. One example is provided by *Melittobia* wasps, where females of several species produce approximately 2% male offspring when ovipositing alone (*n* = 1), and hardly change their offspring sex ratio when more females lay eggs on a patch (larger; Fig. 1A; Abe et al. 2003, 2014). Another example is provided by *Sclerodermus* wasps, in which multiple females can lay eggs on a host but the females still only produce 7% males (Fig. 1B; Tang et al. 2014; Lupi et al. 2017; Abdi et al. 2020a,b,c; Jucker et al. 2020). These cases therefore suggest that we need to identify additional factors that can favor female‐biased sex ratios.

A possible explanation for the observed female biases is that there is the potential for mutually beneficial cooperative interactions between females (Schwarz 1988; Stark 1992; Komdeur et al. 1997; Cronin and Schwarz 1997; Schwarz et al. 1998; Martins et al. 1999; Clutton‐Brock 2002; Tang et al. 2014; Kapranas et al. 2016). For example, in presocial, allodapine bee *Exoneura bicolor*, cooperative nesting occurs among related females, which results in higher per capita reproductive outputs (Schwarz 1988; Cronin and Schwarz 1997). In this case, a more female‐biased sex ratio can be favored to increase these beneficial interactions between related females, as a form of local resource enhancement (LRE; in this literature, we focus on LRE provided from females). Cooperative interactions between females have been suggested to be important in both *Melittobia* and *Sclerodermus* wasps (Abe et al. 2003, 2014; Tang et al. 2014; Lupi et al. 2017). In *Melittobia* wasps, multiple females aggregate on a host (the larvae and pupae of solitary wasps and bees) to co‐parasitize them (J. Abe, unpubl. ms.; Rosenheim 1990), females fight against symbiont mites of host species (Okabe and Makino 2008), and female offspring jointly tunnel into the materials of host nests to disperse (Deyrup et al. 2005). These suggest various types of cooperative interactions between females that could increase female reproductive success. In *Sclerodermus*, the availability of their hosts to ovipositing females positively correlates with the sizes of the hosts, but communal colonization may allow the females to parasitize more successfully and thus to produce more offspring (Tang et al. 2014; Abdi et al. 2020a,b,c). Tang et al. (2014) and Abdi et al. (2020a,b,c) hypothesized that the extremely female‐biased sex ratios in *S. harmandi* may result from LRE. However, the extremely female‐biased sex ratios under LRE in these species remain to be formally explained.

We expand existing theory to examine whether LRE can explain the extremely female‐biased sex ratios that have been observed in *Melittobia* and *Sclerodermus* wasps. We examine three factors that may be especially relevant to the biology of these species: (1) competitions between sons and between daughters; (2) the cooperative interactions can occur at different times, either when adult females produce offspring (i.e., daughters help mothers before they disperse) as in Pen and Weissing (2000) and Wild (2006), or when colonizing females help each other before competition (i.e., offspring help siblings after dispersal). We also consider (3) both females and males may disperse to different extents (sex‐specific dispersal), hence varying the degree to which these competitive and cooperative interactions occur locally. We specifically assess how the sex‐specific dispersal rates, the number of foundresses, and a fecundity effect of LRE jointly influence the evolution of sex ratios.

## Methods

### LIFE CYCLE

**Figure 2:**
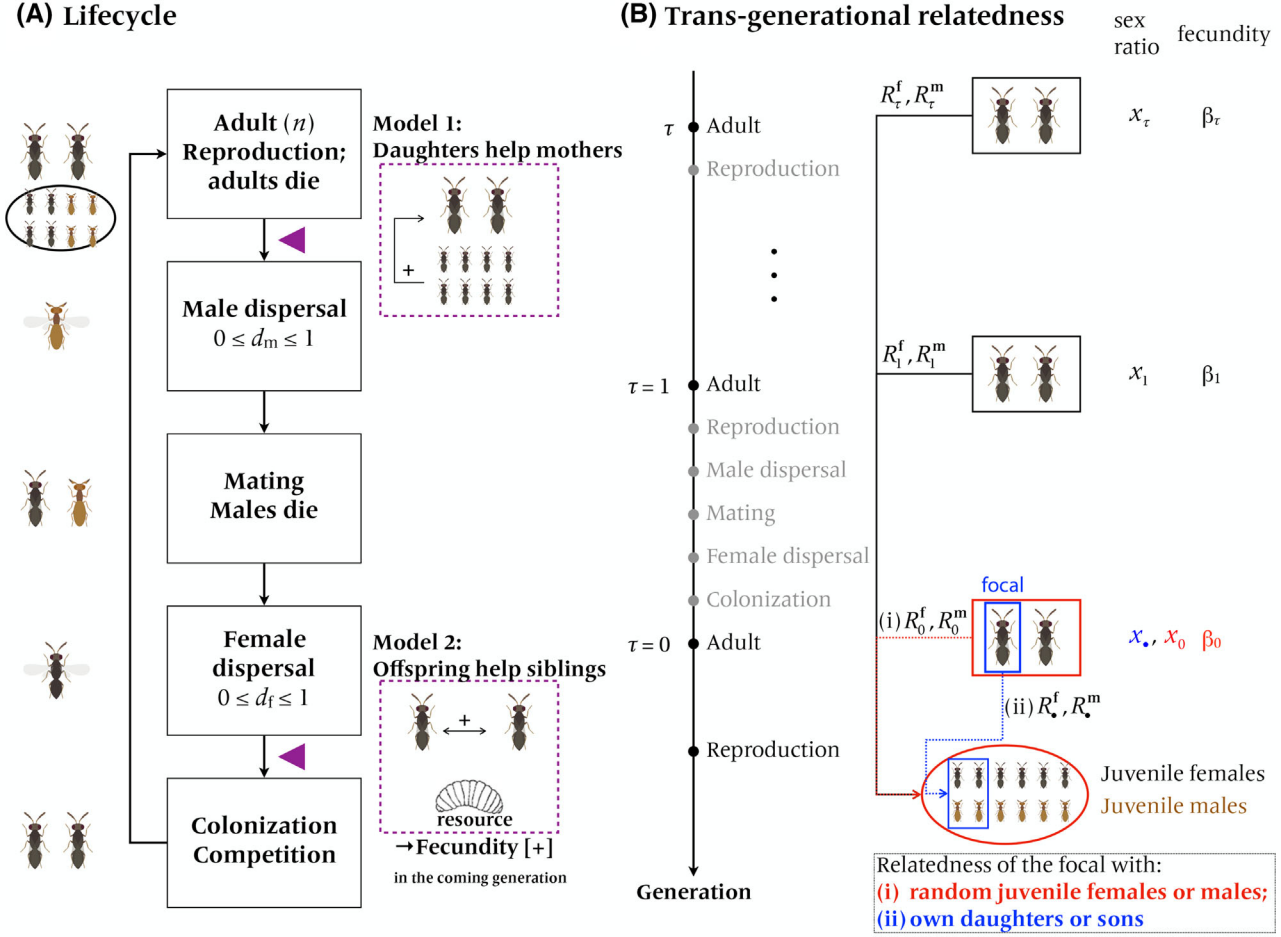
Schematic illustration of (A) lifecycle and (B) relatedness across generations. (A) Gray individuals: females. Brown individuals: males. Purple triangle: possible timing of LRE (before dispersal versus after dispersal). Model species: *Melittobia australica* (but note that males of this species really are flightless; Matthews et al. 2009). (B) The adult within the blue box: the focal individual; the juveniles within the blue box: the focal individual's offspring; red: average in the patch. We count the generations backward in time (*τ* = 0 the present, *τ* = 1 parental, etc). *R*s are relatedness coefficients, each from the corresponding actor's perspective (arrows).

We assume Wright's (1931) island model of dispersal, in which the metapopulation is subdivided into an infinite number of patches each fostering *n* mated females. We focus on a particular female, and denote her proportional investment of reproductive resource into sons (“sex ratio”) by *x*_•_, the average sex ratio of the adult females in her patch in the same generation by $x_{0}$, and the average sex ratio of adult females in the metapopulation by *x*. Immediately upon birth, juvenile males may disperse to an alternative patch at a rate $d_{m}$ each, or else stay in the natal patch 1–*d*_m_, followed by random mating on the patch, with each female mating only once but each male potentially mating many times. Males die after mating and females disperse with a probability of *d*_f_ each. After dispersal, mature females compete for the limited number of breeding sites on the patch (*n*), after which the metapopulation is returned back to its original size and a new cycle starts (Fig. 2A). We use “: =” to define a quantity henceforth. The list of symbols is encapsulated in Table 1.

**Table 1:** Symbols used in the main text

Symbol	Definition	Note
*n*	Patch size (the number of adult females inhabiting a patch).	
*τ*	Generation (counted backward in time): *τ* = 0 for the current, *τ* = 1 for the parental generations, and so forth.	*τ* ≥ 0.
*x*	Sex ratio (proportion sons): *x*_•_ for a focal adult female in a patch in a focal (present) generation; *x*_0_ for the average value in the same patch in the present generation; *x_τ_* for the average value in the same patch τ‐generations ago; *x* for the average value in the metapopulation; and $\overset{⌢}{x}$ for the candidate of evolutionarily stable strategy.	
*d*_m_	Male dispersal rate.	Before mating.
*d*_f_	Female dispersal rate.	After mating.
	Fecundity (per capita):	
β	β_0_ for individuals in the focal patch in the current generation; β*_τ_* for individuals in the focal patch τ‐generations prior; and β° for a random individual in the metapopulation (as a function of *x*).	
*K*	Fecundity per capita in the absence of local resource enhancement.	*K* ≫ 1.
α	Effect of local resource enhancement on individual fecundity.	0 ≤ α ≤ 1.
*B*()	A function that defines the recurrence between the current (β_0_) and the previous generation's averages of fecundity and trait (*β*_1_, *x*_1_): *β*_0_ = *B*(*β*_1_, *x*_1_); More generally, β*_τ_*_−1_ = *B* (β_τ_, *x*_τ_).	
*W* ^f^	Reproductive success of an adult female via her daughters: $W_{•}^{f}$ for the focal adult female in the focal patch; and $W_{0}^{f}$ for a random female in the same patch.	
*W* ^m^	Reproductive success of an adult female via her sons: $W_{•}^{m}$ for the focal adult female in the focal patch.	
*σ*	Scale of competition parameter: *σ*_RC_ = (1 − *d*_f_)^2^ for local resource competition between females; *σ*_MC_ = (1 − *d*_m_)^2^ for local mate competition between males; and *σ*_MB_ = (1 − *d*_m_) (1 − *σ*_RC_) for mating bonus (local availability of females for mating).	
*c*_f_, *c*_m_	Class reproductive values: *c*_f_ = 2/3 and *c*_m_ = 1/3 for haplodiploids, respectively; *c*_f_ = *c*_m_ = 1/2 for haploids and diploids.	
$\hat{x_{\emptyset }}$	Candidate ESS (cESS) for the sex ratio for *α* = 0 (no LRE).	
$R_{•}^{f},\;R_{•}^{m}$	Relatedness of the focal adult female to her daughters and sons, respectively.	
$R_{0}^{f},\;R_{0}^{m}$	Relatedness of the focal adult female to one of juvenile females or males, respectively, born in the same patch in the present generation.	
κ	Scaled relatedness in reference to the expected strength of kin competition.	

We consider two types of LRE. In the first, LRE occurs due to helping behaviors among juvenile females (before dispersal) that promote the survival rate of all juveniles born in the same patch. We refer to this situation as “daughters help mothers before dispersal” in which the fecundity of the adult females in a patch depends on the average sex ratio, *x*_0_, of the focal generation, *τ* = 0 (where we designate *τ* ≥ 0 for a generic symbol to count the generations backward in time: *τ* = 0 for the present, *τ* = 1 for the parental generation, and generally *τ* for *τ*‐generations prior, and we refer to “*τ*‐th generation” henceforth). This model may be relevant in species where juvenile females engage in helping behaviors before dispersal. In the second model, we posit that LRE occurs due to mutual helping at the colonization stage (before competition for breeding spots), the situation in which offspring help siblings after dispersal. This applies to species where females communally colonize common patches, as in *Sclerodermus*.

### MODEL 1: DAUGHTERS HELP MOTHERS BEFORE DISPERSAL

We start with our analyses for the case in which LRE is provided from daughters to mothers before juvenile females disperse, that is, juvenile females help adult females (including the parent of own) producing offspring. We assume that, for a patch with the average sex ratio X, the per capita fecundity (which is the number of offspring born times offspring survival rate) is given by β(*X*) (*X* is used only here). Turning our attention to the focal patch with its inhabitants’ average sex ratio $x_{0}$, female's per capita fecundity (the total number of offspring produced per capita times their survival rate) in the patch is given by $\beta_{0}=\beta \left(x_{0}\right)$. Assuming that the mutants are vanishingly rare with the metapopulation‐wide average sex ratio denoted by *x*, the average fecundity in the metapopulation is given by $\beta^{\circ }:=\beta \left(x\right)$. Using a parameter *α* (with 0 ≤ *α* ≤ 1) that tunes the strength of LRE on fecundity in the focal patch ($\beta_{0}$), we formulate β by

(1a) $$\;\beta^{\circ }=\;\beta \left(x\right)=\;K+\alpha \left(1-x\right)\beta \left(x\right)=\frac{K}{1-\alpha \left(1-x\right)},$$

(1b) $$\;\beta_{0}=\;\beta \left(x_{0}\right)=\frac{K}{1-\alpha \left(1-x_{0}\right)}.$$

(see Appendix A in the Supporting Information for derivation), where *K* is a baseline of per capita fecundity in the absence of LRE. β_0_ is larger when neighboring individuals produce more females (*x*_0_ lower). The fecundity β(*x*_0_) decreases from *K*/(1 − α) to *K* as *x*_0_ varies from 0 to 1, and grows from *K* to *K*/*x*_0_ as α varies from 0 to 1 (for *x*_0_ > 0 fixed).

### MODEL 2: OFFSPRING HELP SIBLINGS AFTER DISPERSAL

We now turn our attention to the case in which LRE is provided from offspring to siblings after dispersal, where juvenile females of the same generation can cooperate after dispersal for communal colonization. We use the same symbol (β_0_) to designate the fecundity of individuals in the focal patch, to keep the consistency with the previous analyses. We write *x_τ_* for the average sex ratio of adult females in the focal patch in the *τ*‐th generation, and β*_τ_* for their fecundity (Fig. 2B). We recursively define β*_τ_* by

(2) $$\begin{array}{ccc} \;\beta_{\tau } & = & \;B\left(\beta_{\tau +1},x_{\tau +1}\right)\\ & = & K+\left(\alpha \left(1-d_{f}\right)\left(1-x_{\tau +1}\right)\beta_{\tau +1}+d_{f}\;\left(1-x\right)\bar{\beta }\right), \end{array}$$

where *B* (,) defines the recurrence relation of the present fecundity per capita (β_0_) with the past fecundity per capita (β_1_; the first argument) and the average sex ratio of the parental generation (*x*_1_; the second argument). Also, α (with 0 ≤ α ≤ 1) measures the strength of LRE as before; (1 − *d*_f_)(1 − *x_τ_*_+1_)β_τ+1_ + *d*_f_ (1 − *x*)β° is proportional to the density of females after female dispersal (before competition); and β° is the metapopulation‐wide average of β to be determined: assuming that it has reached a stable equilibrium value for a phenotypically monomorphic population with *x*, the equilibrium value for β = β° is given as the solution to β° = *B*(β°, *x*); that is,

(3) $$\;\beta^{\circ }=\beta \left(x\right)=\frac{K}{1-\alpha \left(1-x\right)},$$

(see eq. 1), which is always locally stable for given *x* (i.e., β*_τ_* converges to β° given *x* is fixed). Specifically, the fecundity of the focal female in the present generation *τ* = 0 is given by

(4) $$\begin{array}{ccc} \;\beta_{0} & = & \;B\left(\beta_{1},x_{1}\right)\\ & = & \;B\left(B\left(\beta_{2},x_{2}\right),x_{1}\right)=\;B\left(B\left(B\left(\beta_{3},x_{3}\right),x_{2}\right),x_{1}\right)=..., \end{array}$$

(see Appendix A in the Supporting Information for more details), which implies that to determine (the effect of selection on) β_0_, we need to consider an expected sequence of retrospective sex ratios in the focal patch, (*x*_1_, *x*_2_, *x*_3_, …), in addition to the focal's and neighbors’ sex ratios in the present generation, (*x*_•_, *x*_0_) (Lehmann 2007, 2008). LRE supplied from offspring to siblings after dispersal hence generates the transgenerational kin selection effects in viscous populations (i.e., limited dispersal causing local interactions including kin competition), by which the impacts of biased sex ratios in the patch descend down to the reproductive success of individuals (including the focal's offspring) living in future generations, which thus in turn induces selection on the sex ratios.

### INVASION FITNESS AND THE SELECTION GRADIENT

We can write the invasion fitness of the focal female through daughters and sons (respectively) as

(5) $$\begin{array}{ccc} W_{•}^{f}: & = & W^{f}\left(x_{\cdot },x_{0},\beta_{0}\right)\\ & = & \frac{\left(1-d_{f}\right)\left(1-x_{•}\right)\beta_{0}}{\left(1-d_{f}\right)\left(1-x_{0}\right)\beta_{0}+d_{f}\left(1-x\right)\beta^{\circ }}+\frac{d_{f}\left(1-x_{•}\right)\beta_{0}}{\left(1-x\right)\beta^{\circ }}, \end{array}$$

(6) $$\begin{array}{ccc} W_{•\;}^{m}: & = & W^{m}\left(x_{•},x_{0},W_{0}^{f},\beta_{0}\right)\\ & = & \frac{\left(1-d_{m}\right)x_{•}\beta_{0}}{\left(1-d_{m}\right)x_{0}\beta_{0}+d_{m}x\beta^{\circ }}\;\underset{=:W_{0}^{f}}{\underset{︸}{W^{f}\left(x_{0},x_{0},\beta_{0}\right)}}+\frac{d_{m}x_{•}\beta_{0}}{x\beta^{\circ }}, \end{array}$$

(see Lehmann 2007; Gardner et al. 2009; see Appendices B1−3 for derivation), where the invasion subcomponent for sons (eq. 6) is envisioned as a function of the focal adult female's sex ratio *x*_•_, patch‐average sex ratio *x*_0_, the survival rate of a random female as a mate for local males $W_{0}^{f}$ (local mating bonus; see Frank 1998, p. 199), and the average fecundity of the focal adult female β_0_. Note that β_0_ depends on the types of the LRE: equation (1) or (3).

We use the evolutionary invasion analyses (Hofbauer and Sigmund 1990; Dieckmann and Law 1996; Geritz et al. 1998) and carry out the neighbor‐modulated fitness approach to kin selection methodology (Taylor and Frank 1996; Frank 1998; Rousset and Billiard 2000; Rousset 2004; Taylor et al. 2007), particularly for sex‐structured populations (Taylor 1990; Taylor et al. 2007; Gardner et al. 2009) with transgenerational effects of kin selection (Lehmann 2007, 2008). We take a random juvenile female and male in the present generation each as a recipient, and adult females breeding in the *τ*‐th generation (with *τ* = 0, 1, 2, …, including the focal juveniles’ mother) each as an actor.

To ease biological interpretation, we here posit that *σ*_RC_: = (1 − *d*_f_)^2^ tunes the intensity of LRC, which represents the probability that the focal adult female's daughters compete for resources with a juvenile female born in the same patch (equations (7) and A20 in Wild and Taylor 2004). Similarly, the intensity of LMC is proportional to *σ*_MC_: = (1 − *d*_m_)^2^, which is the probability that the focal adult female's sons compete for mates with a juvenile male born in the same patch. Increasing *σ*_RC_ (or *σ*_MC_) favors less (or more) female‐biased sex ratios (respectively). Also, the effect of extra daughters born locally on males’ reproductive success is given by *σ*_MB_: = (1 − *d*_m_)(1 − *σ*_RC_) (i.e., local mating bonus; see Frank 1998, p. 199), which reads as the probability that males mate locally (1 − *d*_m_) times the probability that the females having mated with him do not encounter local resource competition with a juvenile female born in the same patch 1 − (1 − *d*_f_)^2^ = 1 − *σ*_RC_.

Using these *σ*_RC_, *σ*_MC_, and *σ*_MB_, the condition for which a slightly larger sex ratio (i.e., producing more sons than does the metapopulation average) is favored by natural selection is captured by Hamilton's rule:

(7) $$\begin{array}{ccc} & & c_{f}\left(\underset{\mathrm{daughters}}{\underset{︸}{\frac{-1}{1-x}R_{•}^{f}}}+\underset{\mathrm{LRC}\;\mathrm{effect}}{\underset{︸}{\frac{\sigma_{\mathrm{RC}}}{1-x}R_{0}^{f}}}+\underset{\mathrm{LRE}\;\mathrm{on}:\;\mathrm{daughters}\;\&\;\mathrm{LRC}}{\underset{︸}{\sum_{\tau =0}^{+\infty }\frac{1-\sigma_{\mathrm{RC}}}{\beta^{\circ }}\cdot \frac{\partial \beta_{0}}{\partial x_{\tau }}R_{\tau }^{f}}}\right)\\ & & \;+c_{m}\left(\underset{\mathrm{sons}}{\underset{︸}{\frac{1}{x}R_{•}^{m}}}+\underset{\mathrm{LMC}\;\mathrm{effect}}{\underset{︸}{\frac{-\sigma_{\mathrm{MC}}}{x}R_{0}^{m}}}+\underset{\mathrm{Mating}\mathrm{Bonus}\left(\mathrm{MB}\right)}{\underset{︸}{\frac{-\sigma_{\mathrm{MB}}}{1-x}R_{0}^{m}}}\right)\\ & & \;+c_{m}\left(\underset{\mathrm{LRE}\;\mathrm{on}:\;\mathrm{sons}\;\&\;\mathrm{LMC}}{\underset{︸}{\sum_{\tau =0}^{+\infty }\frac{1-\sigma_{\mathrm{MC}}}{\beta^{\circ }}\cdot \frac{\partial \beta_{0}}{\partial x_{\tau }}R_{\tau }^{m}}}+\underset{\mathrm{LRE}\;\mathrm{on}\mathrm{MB}}{\underset{︸}{\sum_{\tau =0}^{+\infty }\frac{\sigma_{\mathrm{MB}}}{\beta^{\circ }}\cdot \frac{\partial \beta_{0}}{\partial x_{\tau }}R_{\tau }^{m}}}\right)>0 \end{array}$$

(based on Taylor's [1981] approach; see also Appendices B4−7 for derivation; Lehmann 2007, 2008), where each derivative is evaluated at phenotypic neutrality (*x*_•_ = *x*_0_ = *x*_1_ = … = *x*). In equation (7), $R_{•}^{f}$ (or $R_{•}^{m}$) represents the regression coefficient of relatedness (“relatedness” hereafter; Michod and Hamilton 1980; Grafen 1985) for a juvenile female (or juvenile male) from the perspective of their mother each in the present generation (subscript *τ* = 0); $R_{0}^{f}$ (or $R_{•}^{m}$) represents the relatedness for a random juvenile female (or juvenile male) from the perspective of a random adult female in the same patch each in the present generation; and $R_{\tau }^{f}$ (or $R_{\tau }^{m}$) represents the relatedness for a random juvenile female (or juvenile male) in the present from the perspective of an adult female in the *τ*‐th generation (Taylor 1988; Bulmer 1994); *c*_f_ (or *c*_m_) represents the class reproductive value of female (or male; Taylor 1990; Caswell 2001; the probability that a randomly sampled allele from the gene pool is found in an individual of the corresponding sex). In this article, we consider haplodiploids to set *c*_f_ = 2/3, *c*_m_ = 1/3, but our calculation equally applies to diploids by substituting *c*_f_ = *c*_m_ = 1/2. We again remark that we use different β_0_ depending on the types of LRE.

In the absence of LRE (α = 0, thus the summation Σ terms in eq. 7 vanishing), investing maternal reproductive resources into sons has five consequences: the decrease in daughters’ success, decrease in LRC, increase in sons’ success, increase in LMC, and decrease in MB, as in the previous studies (Taylor 1981). The summation (∑) terms capture the sum of LRE effects each supplied by the individuals having colonized the focal patch at time epochs *τ* = 0 (for the LRE provided from daughters to mothers), and *τ* = 1, 2, … (for the LRE provided from offspring to siblings), on the focal female's fitness (Fig. 2B); this inclusive fitness effect occurs by which (i) LRE increases the number of sons and thus LMC, (ii) LRE increases the number of daughters and LRC, and (iii) LRE increases MB for sons.

Nullifying and solving equation (7) for *x* yields a candidate ESS of sex ratio (cESS henceforth; Christiansen 1991; Takada and Kigami 1991), which we generically designate with a hat ($\hat{x}$).

## Results

### NO LRE

**Figure 3:**
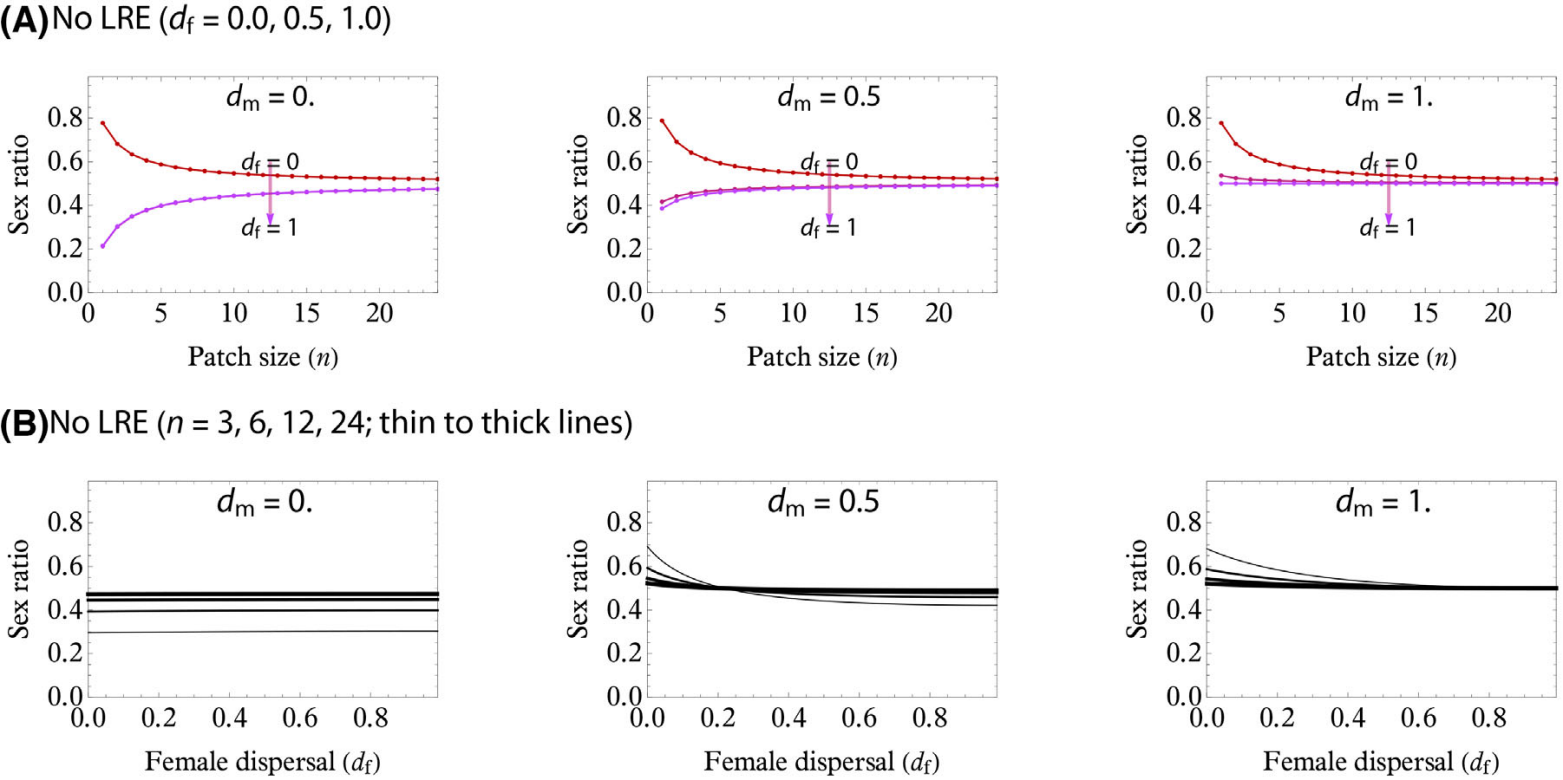
Evolutionary outcomes of sex ratio without LRE under haplodiploidy. (A) Increasing *n* monotonically favors Fisherian sex ratio. Increasing *d*_f_ is likely to favor a less male bias. Note that *d*_f_ = *d*_m_ = 0 (red curve in the left panel) is an exceptional case in which all patches are mutually isolated, and this case therefore invalidates the present analyses (instead, entailing stochastic analyses). For (slightly) positive values for *d*_f_ > 0, the evolutionary outcomes show very weak sensitivity to female dispersal rate (purple curves heavily overlapped in the left panel, which are visually difficult to separate from each other). (B) Dependence on *d*_f_ (with *d*_m_ = 0, 0.5, 1.0 from left to right panels, and *n* = 2, 4, 8, 16 from thin to thick curves). Generally, high group sizes (*n*) favor Fisherian sex ratio. When male dispersal is completely limited (left panel), sex ratio is almost invariant with *d*_f_. Increasing *d*_m_ results in a shift to male‐bias when female dispersal is small, and as *d*_f_ increases, the female bias is likely to be favored by selection (middle). When male dispersal is complete *d*_m_ = 1, the resulting sex ratio is male‐biased and approaches Fisherian (1/2) as *d*_f_ increases. All figures produced by nullifying equation (7).

We first assess the case for α = 0 (no LRE). By nullifying equation (7) for $\hat{x_{\emptyset }}$ with α = 0 gives

(8) $$\;\hat{x_{\emptyset }}=\frac{c_{m}\left(R_{•}^{m}-\sigma_{\mathrm{MC}}R_{0}^{m}\right)}{c_{m}\left(R_{•}^{m}-\left(\sigma_{\mathrm{MC}}-\sigma_{\mathrm{MB}}\right)R_{0}^{m}\right)+c_{f}\left(R_{•}^{f}-\sigma_{\mathrm{RC}}R_{0}^{f}\right)},$$

in agreement with Wild and Taylor (2004). From equation (8) we can assess how local interactions jointly determine the evolutionary outcomes of sex ratios; we remark that *σ*_RC_ = (1−*d*_f_)^2^, *σ*_MC_ = (1−*d*_m_)^2^, and *σ*_MB_ = (1−*d*_m_)(1−*σ*_RC_). Also, notice that for males, the total scale of competition, which includes the effect of LRC among the males’ mates (i.e., females that thus received males’ gametes), reads *σ*_MC_ − *σ*_MB_, which is negative when (1 − *d*_f_)^2^ < *d*_m_ < 1 (null for either *d*_m_ = 1 or *d*_m_ = (1 − *d*_f_)^2^; otherwise positive). Equation (8) is a general expression of cESS under LRC and LMC (but without LRE) when male dispersal precedes mating and subsequent female dispersal (dispersal‐mating‐dispersal model, or “DMD model”, in Wild and Taylor 2004). Substituting equilibrium values of the relatedness shows that $\hat{x_{\emptyset }}$ exhibits overall a female or male bias when *d*_m_ is small or large (respectively) and it approaches 1/2 (Fisherian sex ratio) as *n* increases (Fig. 3A).

As in the classical LMC theory, inserting *d*_m_ = 0 (no male dispersal as in *Melittobia*; Matthews et al. 2009) yields *σ*_MC_ = 1(≥ *σ*_RC_) and *σ*_MB_ = 1 − *σ*_RC_, meaning that *σ*_MC_ − *σ*_MB_ equals *σ*_RC_ (see the denominator of eq. 8); this subsequently supplies

(9) $$\;\hat{x_{\emptyset }}{|}_{\;d_{m}=0}=\frac{c_{m}\left(R_{•}^{m}-R_{0}^{m}\right)}{c_{m}\left(R_{\;•}^{m}-\sigma_{\mathrm{RC}}R_{0}^{m}\right)+c_{f}\left(R_{•}^{f}-\sigma_{\mathrm{RC}}R_{0}^{f}\right)},$$

(Appendix B7 in the Supporting Information; eq. 3 in Gardner et al. 2009). Particularly for haploids and diploids, we get the well‐known formula $\hat{x_{\emptyset }}$ = (*n* − 1)/(2*n*) regardless of the female dispersal rate (Hamilton 1967; Bulmer 1986; Frank 1986b; Taylor 1988; Bulmer 1994; Frank 1998; Gardner et al. 2009). This dispersal‐invariance is due partly to the concomitant effects of producing more daughters on weaker LMC but stronger LRC with these effects exactly canceling one another out (Taylor's [1992] cancelling principle; Wilson et al. 1992; Taylor 1992). For haplodiploids, equation (8) certainly depends on the female dispersal rate but in a negligibly minor manner (almost‐invariance in female dispersal; Fig. 3B, left panel).

In the case for *d*_m_ > 0, male‐biased sex ratios may occur when *d*_f_ is small, in contrast to the almost‐invariance result for *d*_m_ = 0 (Fig. 3B). Increasing *d*_m_ > 0 (say 0.5) leads to strongly male‐biased sex ratios yet with a possibility of female bias when *d*_f_ is relatively large (Fig. 3B). When *d*_m_ = 1 (thus *σ*_MC_ = *σ*_MB_ = 0), the male bias is always selected for ($\hat{x_{\emptyset }}$ > 1/2; Fig. 3B; see also figure in Appendix C in the Supporting Information). Overall, we find that sex ratio tends to bias toward the more dispersing sex, consistent with Bulmer and Taylor's (1980), Taylor's (1994), and Wild and Taylor's (2004) predictions.

### EFFECTS OF LRE: GENERAL PATTERNS

**Figure 4:**
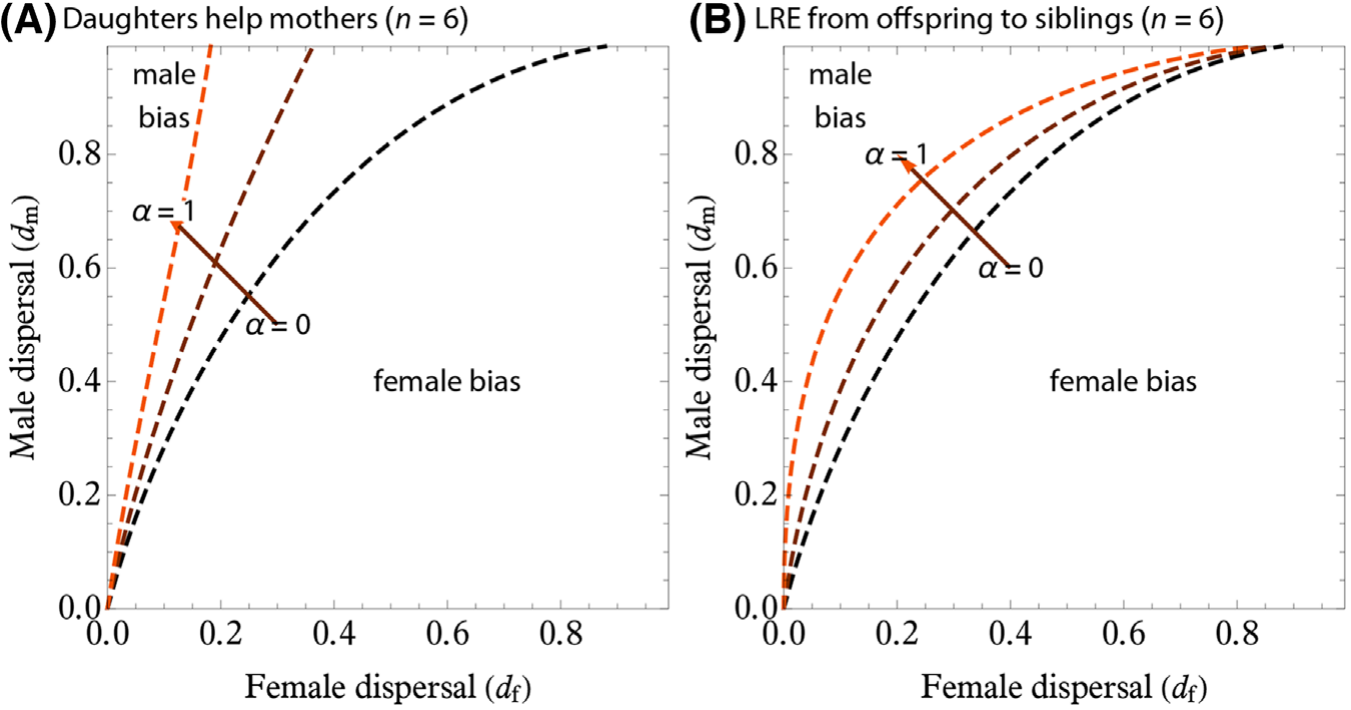
Threshold conditions for the biased sex ratios at cESS (i.e., contours for $\hat{x}=1/2$). Each contour represents the condition for Fisherian sex ratio (1/2) to be cESS and separates the region for female‐ and male‐biased sex ratios. Female‐biased sex ratios (bottom right zones) become more likely as α increases (where threshold curves plotted for α = 0, 0.5, and 1). The contours are produced using equation (7) with *x* = 1/2 (Fisherian sex ratio) inserted.

We now consider the consequences of LRE (α > 0). We found three general patterns. First, both types of LRE favor more female‐biased sex ratio and less male‐biased sex ratios (Fig. 4A; see also Fig. S1). Second, the effect of LRE supplied by daughters to mothers is stronger than that of LRE after dispersal (Fig. 4B); more precisely, the effect of LRE provided from daughters to mothers is independent of sex dispersal propensities of both sexes, whereas that of LRE provided from offspring to siblings decreases with the dispersal rates of both sexes. Finally, *d*_m_ = 0 (no male dispersal) as in the classic LMC theory leads to “almost‐invariance results”, in which cESS sex ratio is insensitive to female dispersal rate (Hamilton 1967; Bulmer 1986; Frank 1986b; Taylor 1988; Bulmer 1994; Frank 1998; Gardner et al. 2009).

### MODEL 1: DAUGHTERS HELP MOTHERS BEFORE DISPERSAL

We deal with general values of dispersal rates (*d*_f_ and *d*_m_, each ranging between 0 and 1), but will make an exception for *d*_m_ = 0 (no male‐dispersal), because the results for *d*_m_ = 0 are qualitatively different from the results for general values 0 < *d*_m_ ≤ 1. The other advantage of presenting the specific result for *d*_m_ = 0 is that this assumption gives a simple formula, comparable with the previous theoretical work (Hamilton 1967; Bulmer 1986; Frank 1986b; Taylor 1988; Bulmer 1994; Frank 1998; Gardner et al. 2009), and applies to many species such as *Melittobia* (Matthews et al. 2009). As such, we present the results for *d*_m_ = 0 and 0 < *d*_m_ ≤ 1 separately; note that *d*_m_ = *d*_f_ = 0 means that patches are completely isolated from each other and entails stochastic analyses (Sigmund 2010), and so we omit this possibility.

We find that Hamilton's rule (eq. 7), which assesses the direction of selection, is equal to

(10) $$\hat{x_{\emptyset }}-x-\underset{\mathrm{scaled}\;\mathrm{relatedness}}{\underset{︸}{\kappa }}\cdot \frac{\alpha \left(1-x\right)}{{\underset{︸}{1-\alpha \left(1-x\right)}}_{\mathrm{LRE}\;\mathrm{effect}\;\left(>0\right)}}\cdot x>0,$$

where *κ*, referred to as “scaled relatedness,” measures the extent to which the extra juveniles produced via LRE are likely to share the common ancestor (see Appendix B in the Supporting Information for more precise interpretation and expression; eq. S36), as a function of *n*, *d*_f_, and *d*_m_, in reference to the expected strength of kin competition (van Cleve 2015). The last term represents the effect of LRE provided from daughters to mothers on the inclusive fitness of the focal individual. Clearly, with LRE, cESS is smaller than $\hat{x_{\emptyset }}$, that is, LRE leads to more female‐biased sex ratios (Fig. 4A).

**Figure 5:**
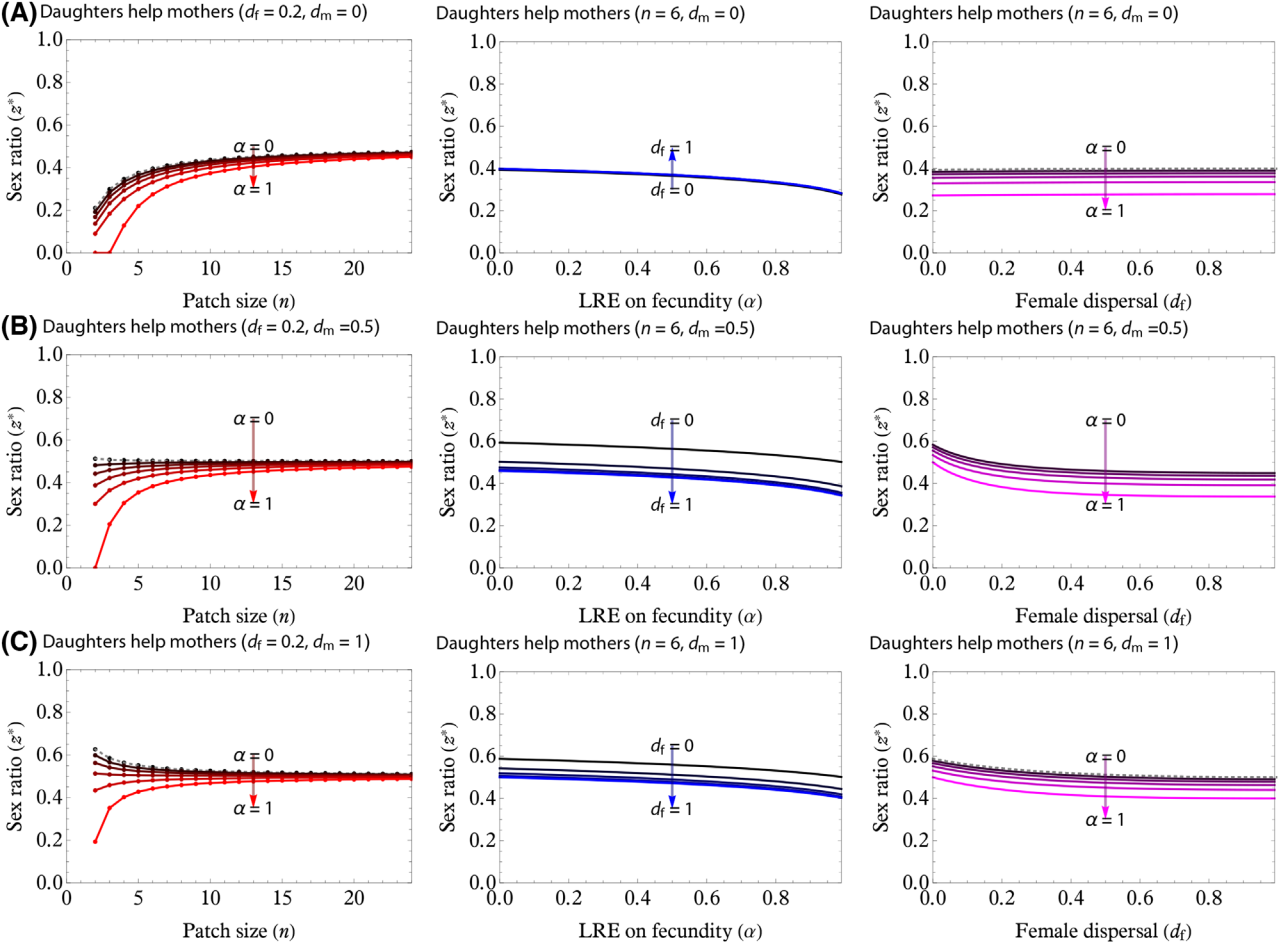
cESS for the LRE provided from daughters to mothers (the first model). Male‐dispersal rate *d*_m_ is varied from (A) 0.0, (B) 0.5, and (C) 1.0. Female dispersal rate is fixed *d*_f_ = 0.2 in each panel, and the other parameter values are as indicated. All panels are produced by nullifying equation (7). Left panels: cESS is plotted against the patch size *n*, with the intensity of LRE, α, increased from 0.0 to 1.0 in 0.2 increments. Note that gray dots are the results for α = 0 ($\hat{x_{\emptyset }}$; eq. 8). When the male‐dispersal rate is small, the cESSs tend to be female biased, but the increase in the male‐dispersal rate may cause cESSs to be male‐biased. In either case, increasing the intensity of LRE (α) can favor lower values of cESS and thereby causes less male‐biased or more female‐biased sex ratios. Middle panels: cESS is plotted against α for 0 ≤ α ≤ 1. Increasing α leads to lower values of cESS. Right panels: cESS is plotted against *d*_f_. When (A) *d*_m_ = 0, the cESS exhibits negligibly small dependence on *d*_f_, and this trend occurs when we consider LRE (α, in 0.2 increments from 0 to 1). When *d*_m_ is (B) at an intermediate value (*d*_m_ = 0.5) or (C) very high (*d*_m_ = 1), small values of *d*_f_ likely predict male‐biased sex ratios to be cESS, but increasing α may result in female bias.

We numerically evaluated the cESS to find that larger group sizes favor less female‐biased sex ratio and the cESS eventually approaches 1/2 (or Fisherian sex ratio) as *n* → +∞ (Fig. 5, left panels). Increasing α leads to more female‐biased sex ratios (Figs. 4 and 5). As in the results for no LRE, sex ratios may be biased toward the more dispersing sex, but LRE causes the evolution of female‐biased sex ratios to be more likely.

#### Example: no male‐dispersal, d_*m*_ = *0*

Suppose for now *d*_m_ = 0, and in this case, we can show that *κ* = 1/*n* (Taylor 1992; Gardner et al. 2009) and therefore ploidy has no influence on the effect of LRE or *κ* (Taylor 1992; Lehmann 2007). This is partly because males are fully philopatric, mating takes place in prior to female dispersal, and female dispersal allows males’ and females’ gametes both to disperse by the same degree, which leads to *σ*_MC_ − *σ*_MB_ = (1 − *d*_f_)^2^ ≡ *σ*_RC_, that is, males and females are subject to the same degree of local competition, where “≡” is identity (“always equivalent to”). This scenario is similar to plants undergoing gametic (pollen) and zygotic (seed) dispersal when pollen dispersal is fully restricted within a patch (see Rousset 2004; Ravigné et al. 2006; Iritani 2020 for more details). That is, decomposing the scale of competition tells us otherwise missed fact: when *d*_m_ = 0, the scale of competition for both sexes is equivalent, thereby generating the almost‐invariance result.

#### Varying male‐dispersal, d_*m*_ > *0*

Now we tune *d*_m_ from 0 to 1 and assess its impacts upon cESS so that we can clarify why the case *d*_m_ = 0 makes an exception. We find that increasing *d*_m_ or *d*_f_ is likely to favor less or more female‐biased sex ratios (Fig. 5; respectively), and taking both to 1 leads to Fisherian sex ratio. For an intermediate male dispersal (*d*_m_ = 0.5), male bias is still likely but with a possibility of switching from male to female bias as α or *d*_f_ increases. Therefore, under the LRE from daughters to mothers, the sex ratios, which could be otherwise male biased, may be biased toward female by natural selection.

### MODEL 2: OFFSPRING HELP SIBLINGS AFTER DISPERSAL

**Figure 6:**
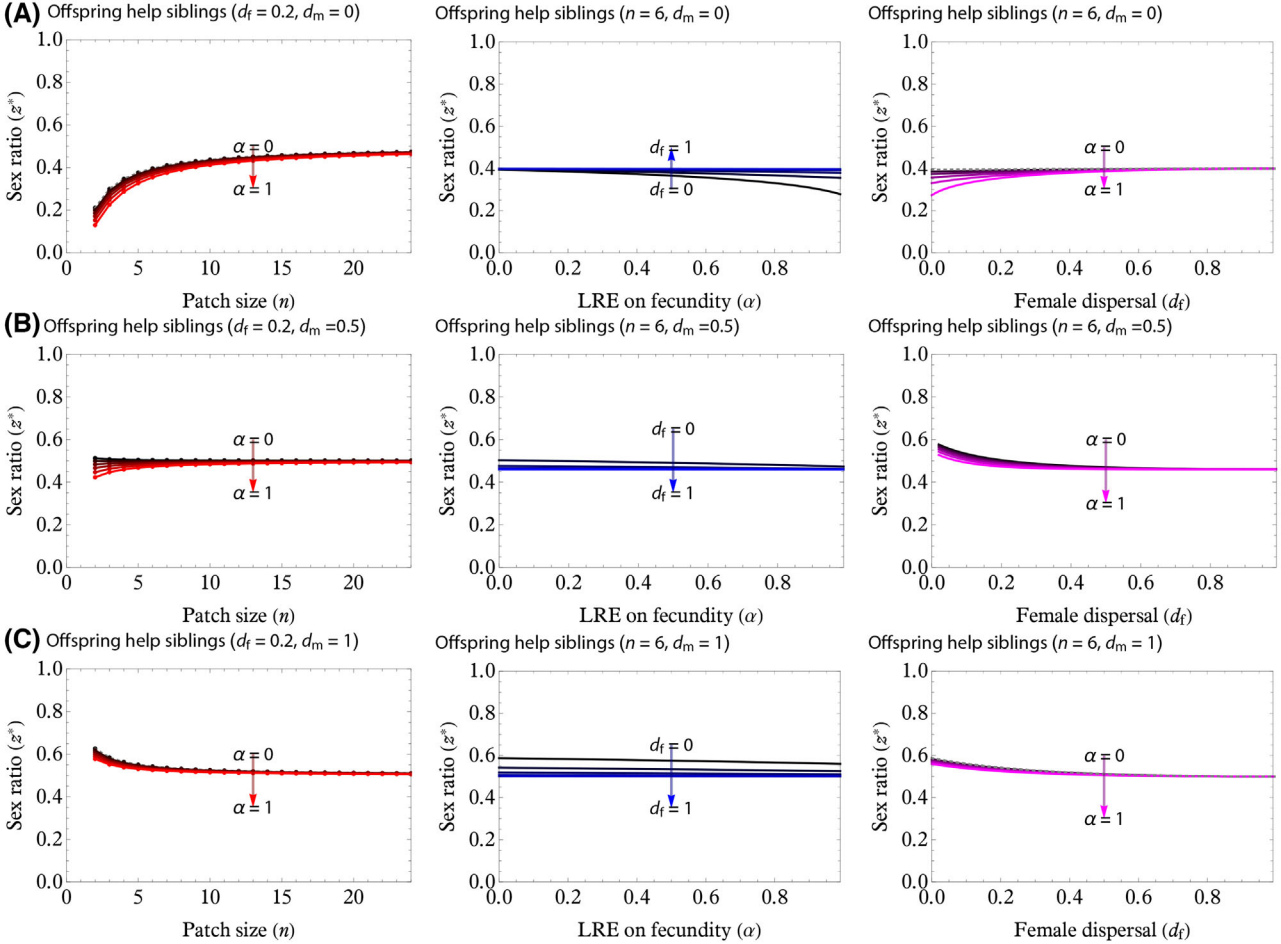
cESS for the LRE provided from offspring to siblings after dispersal (the second model). This figure applies the same scheme as does Figure 5 and is therefore comparable with it. The overall trend is similar to the first model (LRE before dispersal), but the effect of the LRE parameter α is generally weaker than in the first model. As a result, when male dispersal is intermediate (panel B, left panel), increasing α may switch the cESS back from the male to female bias. When male dispersal is large, however, α does not allow for the female‐biased cESS to occur.

We find that LRE from offspring to siblings after dispersal also facilitates the evolution of female‐biased sex ratio (Fig. 4B). Numerical estimation revealed that larger group sizes favor less female‐biased sex ratio and the cESS eventually approaches 1/2 (or Fisherian sex ratio) as *n* → +∞ (Fig. 6, left panels), and increasing α leads to more female‐biased sex ratios (Fig. 6), as in the LRE from offspring to siblings after dispersal. The inclusive fitness effect of the LRE after dispersal decreases with *d*_f_; when *d*_f_ = 1 (full female dispersal), for instance, the transgenerational kin‐selection effect vanishes for any α > 0 (Fig. 6A, middle panel). The LRE that ensues after female dispersal is therefore sensitive to *d*_f_ because the probability that females can help their relatives (by remaining in the natal patch, 1 − *d*_f_) decreases with *d*_f_.

#### Example: No male‐dispersal, *d*_m_ = 0

When *d*_m_ = 0, we find that Hamilton's rule reads

(11) $${\left.\hat{x_{\emptyset }}\right|}_{\;d_{m}=\;0}-x-\frac{1}{n}\cdot \frac{\alpha \sigma_{\mathrm{RC}}\left(1-x\right)}{{\underset{︸}{1-\alpha \sigma_{\mathrm{RC}}\left(1-x\right)}}_{\mathrm{LRE}\;\mathrm{effect}\;\left(>0\right)}}\cdot x>0,$$

where the scaled relatedness is now given by *κ* = 1/*n* as in the model of daughters helping mothers, but the last term in equation (11) is clearly smaller than that in equation (10); the effect of LRE provided from offspring to siblings is therefore weaker than that from daughters to mothers.

#### Varying male‐dispersal, d_*m*_ > *0*

Varying *d*_m_ > 0 turns out to give complicated form of Hamilton's rule (see Appendix B10 in the Supporting Information, eq. S46), except for the extreme case *d*_m_ = 1:

(12) $${\left.\hat{x_{\emptyset }}\right|}_{d_{m}=1}-x-\frac{c_{m}R_{0}^{m}+c_{f}\left(R_{0}^{f}-\sigma_{\mathrm{RC}}R_{0}^{f}\right)}{c_{m}R_{•}^{m}+c_{f}\left(R_{•}^{f}-\sigma_{\mathrm{RC}}R_{0}^{f}\right)}\cdot \frac{\frac{\alpha }{2}\sigma_{\mathrm{RC}}\left(1-x\right)}{{\underset{︸}{1-\frac{\alpha }{2}\sigma_{\mathrm{RC}}\left(1-x\right)}}_{\mathrm{LRE}\;\mathrm{effect}\;\left(>0\right)}}\cdot x>0,$$

which tells us that α*σ*_RC_ in equation (11) is now replaced with α*σ*_RC_/2, with 1/2 meaning that only half of females’ genes are transmitted to females (who, as opposed to males all dispersing, are likely philopatric and thus potentially contribute to the buildup of transgenerational relatedness). From the expression, the scaled relatedness *κ* is now in reference to zero (no LMC nor MB) for males and $\sigma_{\mathrm{RC}}R_{0}^{f}\geq 0$ (LRC, which is zero when all females disperse *d*_f_ = 1) for females.

### EXTENSION: MULTIPLICATIVE FUNCTION OF FECUNDITY

We have so far assumed that the functional forms of LRE are additive (equations 1 and 3). We can consider stronger effects of LRE by using a multiplicative form of LRE, that is,

(13) $$\beta_{0}={\left(\left(1-x_{0}\right)K\beta_{0}\right)}^{\alpha }={\left(\left(1-x_{0}\right)K\right)}^{\frac{\alpha }{1-\alpha }}$$

for the LRE provided from daughters to mothers; Hamilton's rule reads

(14) $$\hat{x_{\emptyset }}-x-\kappa \cdot \underset{\mathrm{LRE}\;\mathrm{effect}\;(>0)}{\underset{︸}{\frac{\alpha }{1-\alpha }}}\cdot x>0$$

(c.f. equation 10). We can thus analytically obtain $\hat{x_{\tau =0}}=\hat{x_{\emptyset }}\left(1-\alpha \right)/\left(1-\alpha +\alpha \kappa \right)$, which converges to $\hat{x_{\tau =0}}\to 0$ as α→1; that is, the effect of LRE is quantitatively much stronger and α ≈ 1 leads to arbitrarily small value of cESS (see Fig. S3).

For the LRE provided from offspring to siblings after dispersal, similarly, we can use a fecundity function of the form

(15) $$\begin{array}{ccc} \beta_{\tau } & = & B\left(\beta_{\tau +1\;},x_{\tau +1}\right)\\ & = & {\left(\left(1-d_{f}\right)\left(1-x_{\tau +1}\right)\beta_{\tau +1}+d_{f}\left(1-x\right)\bar{\beta }\right)}^{\alpha }K^{\alpha }, \end{array}$$

where

(16) $$\bar{\beta }={\left(\left(1-x\right)K\right)}^{\frac{\alpha }{1-\alpha }}$$

From this, Hamilton's rule is obtained as

(17) $$\hat{x_{\emptyset }}-x-\kappa \cdot \underset{\mathrm{LRE}\;\mathrm{effect}\;(>0)}{\underset{︸}{\frac{\alpha \sigma_{\mathrm{RC}}}{1-\alpha \sigma_{\mathrm{RC}}}}}\cdot x>0$$

resulting in $\hat{x_{\tau =0}}=\hat{x_{\emptyset }}\left(1-\alpha \sigma_{\mathrm{RC}}\right)/\left(1-\alpha \sigma_{\mathrm{RC}}+\alpha \sigma_{\mathrm{RC}}\kappa \right).$

Despite the greater consistency of the multiplicative LRE models with the data (Figs. 1 and S3), the assumption of this multiplicative function may be more restrictive than that of additive assumption; specifically, equation (16) implies that (i) when α = 0, the baseline fecundity is equal to unity (not *K*), and (ii) the metapopulation‐wide mean fecundity β decreases nonlinearly with *x* and eventually approaches zero with *x* → 1, in contrast to the case for additive effects of LRE. However, *σ*_RC_ = 0 (full female‐dispersal) results in $\hat{x}=\hat{x_{\emptyset }}$ (no LRE) as in the additive version of LRE (after dispersal). Therefore, the effect of LRE is once again stronger than the additive case but decreases with female dispersal rate as in the additive LRE (Fig. S3).

## Discussion

We found that cooperative interactions between females (LRE) can lead to even more female‐biased sex ratios under conditions of LMC theory. Specifically, we have considered two types of LRE, and found that cooperation from offspring to their parents’ generation can lead to more female‐biased sex ratios than cooperation between members of the same generation (intergenerational LRE). This difference is because we assumed that daughters help mothers before they disperse, and therefore they have direct access to helping genetically related juveniles, thereby increasing the inclusive fitness of the mothers producing daughters over sons. In contrast, after dispersing, dispersed juvenile females are unable to provide help to relatives (as in the LRE model of offspring helping siblings), thereby reducing the inclusive fitness benefit of LRE as *d*_f_ increases, unless females disperse in a group (budding‐dispersal; Avilés 1993; Gardner et al. 2009). However, especially in *Melittobia* wasps, the observed sex ratios are still more female biased than predicted by theory, suggesting that an additional factor is at play (Figs. 1, 5, and 6; see below) Our key result is therefore that LRE, alongside LMC, has the potential to generate more female‐biased sex ratios than predicted from LMC theory alone, confirming the verbal prediction provided in the previous experimental studies (Tang et al. 2014). Our results also allow for quantitative comparisons between the present predictions and data.

As found by previous theory, we showed that, in the absence of LRE (α = 0), natural selection in general favors a sex ratio bias toward the more dispersing sex (Bulmer and Taylor 1980; Taylor 1994; Wild and Taylor 2004), which is because kin competition between members of one sex reduces the inclusive fitness benefits of producing that sex. In contrast, when females interact cooperatively (LRE), this provides an inclusive fitness benefit of producing females, and natural selection thus favors less male‐biased or more female‐biased sex ratios (Figs. 4 and 5; Taylor 1981; Emlen et al. 1986; Pen and Weissing 2000; Wild and Taylor 2004; Wild 2006; Wild and West 2009; but see Khwaja et al. 2017).

Despite the formal similarity between the two LRE models, there is a quantitative difference in the consequences of dispersal rates for sex ratios. If LRE occurs for daughters helping mothers before dispersal, increasing the intensity of LRE (α) leads to more female‐biased sex ratio by increasing the benefit of producing juvenile females who assist their mother (Figs. 5 and 6). This selective force acts even when female dispersal rate is high, because cooperating before dispersal allows juvenile females to assist their own mother. In contrast, the model of LRE provided from offspring to siblings after dispersal predicts that increasing the intensity of LRE (α) has a weaker effect on the selection for the female bias (Figs. 4 and 6) compared to the model of LRE provided from daughters to mothers. The inclusive fitness effect of LRE from offspring to siblings after dispersal vanishes if females undergo complete dispersal; in other words, *d*_f_ = 1 implies that cESS is independent of α. This result is because following complete female‐dispersal, dispersed juvenile females (the proportion *d*_f_) do not have the access to their relatives and they are unable to engage in helping genetic relatives. Hence, the two models suggest that distinguishing the timing of LRE (before or after dispersal) is of crucial importance for sex ratio evolution in empirical and experimental systems.

Our models could help explain the extreme sex ratio biases that have been observed in *Sclerodermus harmandi*, and several *Melittobia* wasp species. In both these cases, LMC is likely, but the offspring sex ratios are much more female biased than would be expected from LMC theory (Fig. 1). We have shown in the model of LRE after dispersal, which is motivated by *Sclerodermus* and *Melittobia* wasps, that a combination of cooperative interactions between sisters (LRE) and LMC can lead to more female‐biased sex ratios. In *S. harmandi*, females cooperate to suppress hosts and engage in brood care (Hu et al. 2012; Tang et al. 2014; Kapranas et al. 2016; Lupi et al. 2017). In addition, it has been suggested that whether wasps cooperate by attacking the preys in a group may be also subject to natural selection (Mesterton‐Gibbons and Hardy, pers. commun.), which may thus generates complicated interactions between sex allocation and cooperation, especially under kin‐recognition in *S. harmandi* (Abdi et al. 2020a,b,c). Our models therefore offer testable predictions for the female‐biased sex ratios in these species, and provide a modeling framework for future studies to combine the joint evolutionary dynamics of sex ratios and LRE.

However, in *Melittobia australica*, the quantitative discrepancy between data and predictions appears to be still large (Figs. 1 and 6). Possible factors may include the stronger effects of LRE on even more female‐biased sex ratios as shown in the multiplicative functional forms, or it may even be suggested that some constraints limit the facultative sex ratios in *M. australica* (Shuker and West 2004; Greeff et al. 2020). Nonetheless, these sex ratios are still more female biased than predicted by our models. Although the effect of LRE favoring more female‐biased sex ratio cab be cancelled out by the effect of competition between interacting females (Bulmer 1986; Frank 1986a; Taylor 1988, 1992; Wilson et al. 1992; Gardner et al. 2009), the degree of the cancelation could be reduced by additional factors such as overlapping generations, inelasticity, dispersing with relatives, and the availability of empty patches (Taylor 1992; Taylor and Irwin 2000; Alizon and Taylor 2008; Gardner et al. 2009). A recent field study found that sex ratios in *M. australica* depend on female dispersal status, and confirms theoretically and experimentally that sex ratios by dispersing females (but not by nondispersing females) increase with foundress numbers (Abe et al. 2020). Future studies are awaited to elucidate the extreme sex ratios in *Melittobia*.

Besides wasps, our models could also be applied to other species where LRE occurs. For instance, the allodapine bee *Exoneura bicolor* provides evidence for LRE via cooperative nesting between related females (Schwarz 1988; Cronin and Schwarz 1997), and the helping from offspring to siblings after dispersal is suggested to occur in neotropical solitary bee, *Diadasina distincta* (Martins et al. 1999). The model of offspring helping siblings after dispersal may therefore explain the female‐biased sex ratios in these bees. Similarly, a social spider *Anelosimus eximius*, in which females (but not males) engage in colony tasks, exhibits female‐biased sex ratios (Vollrath 1986; Frank 1987). In those cases, LRE is suggested to occur between siblings after dispersal. Other animal systems also suggest the occurrence of LRE provided from daughters to mothers, and therefore female‐biased sex ratios in these species may result from the LRE before dispersal (e.g., large carpenter bee, Stark 1992; cooperatively breeding birds, Komdeur et al. 1997; meerkat, Clutton‐Brock 2002; also see Greeff 1999 for an overview of LRE and sex ratios). The present models demonstrate that distinguishing the timing of LRE (e.g., before vs. after dispersal) with their effects making the difference in the strength of LRE on the evolution of female‐biased sex ratios. We finally remark that if LRE ensues after competition for reproduction, the LRE is neutral to the evolution sex ratios; rather, group size *n* may be more important (a form of Allee effects; Allee 1927). Future studies could investigate other life history factors, such as sex‐biased dispersal, mating system—in which the number of matings for females—, and which sex tends to be more cooperative.

To conclude, our analyses suggest that LRE provided from juvenile females promoted female‐biased sex ratios, but the impacts upon the evolutionary outcomes differ in the consequences of female dispersal depending upon whether helping occurs before or after dispersal; LRE before dispersal does not depend on female‐dispersal rate but the effect of LRE occurring after dispersal decreases with female‐dispersal rate. One of the possible extensions of the present model is to study joint evolution of sex ratio and other traits under LRE (Mullon et al. 2016, 2018). For instance, how does joint evolution shape the association between sex‐biased dispersal and sex allocation strategy, for example, in birds and vertebrates (Frank 1990; Komdeur et al. 1997; Goltsman et al. 2005; Banks et al. 2008; Hjernquist et al. 2009)? Also, our models are restricted to the case in which each female mate only once; using the probability of sib‐mating (which is similar to self‐fertilization in plants; Ravigné et al. 2006; Iritani 2020) offers an alternative approach. Future studies could be directed toward more realistic modeling of LRE, by, for example, incorporating the effects of the number of adult females (*n*) on LRE, or working on intrasexual LRE (“who helps whom”; Rodrigues and Gardner 2013; Rodrigues and Kokko 2016). Working with specific organisms of interest with multiple approaches may yield a better understanding of the evolution of sex ratios, or more generally social evolution, in viscous populations.

## AUTHOR CONTRIBUTIONS

JA conceived the idea. RI carried out the mathematical analyses. JA and RI drafted the first version of the manuscript. All authors contributed to the revision.

## DATA AVAILABILITY STATEMENT

No data are used in this study.

Associate Editor: J. Slate

## Supporting information


**Figure S1**: cESS plotted against sex‐dependent dispersal rates.
**Figure S2**: Schematic illustration to develop recursive equations for the consanguinity across generations.
**Figure S3**: cESS when the effect of LRE is of multiplicative function.
