# Supplementary material for: Cooperative interactions among females can lead to even more extraordinary sex ratios
Source: Evol Lett. 2021 Jun 3;5(4):370–84. doi: 10.1002/evl3.217 (PMC8327954; doi:10.1002/evl3.217)
Supplement: Supplementary file 1 — Figure S1: cESS plotted against sex‐dependent dispersal rates. Figure S2: Schematic illustration to develop recursive equations for the consanguinity across generations. Figure S3: cESS when the effect of LRE is of multiplicative function. [file EVL3-5-370-s001.pdf]

Appendix

A Fecundity mediated by LRE

A-1 Derivation

Let us for now focus on the effects of LRE provided from daughters to mothers (before dispersal). We pick a focal patch and denote the average phenotype on that patch by x_0; on that patch, the fecundity per capita is assumed to be the sum of (i) the baseline fecundity (denoted by K), and (ii) the effects of LRE; the latter occurs from helping behaviors of daughters, and the total number of females per patch is given by $n \cdot (1-x_0) \cdot \beta(x_0)$, in which the effect of help from daughters is equally shared by n females and thus should be divided by n (therefore n being canceled), which hence yields:

$$\beta_0 := \beta(x_0) = K + \alpha(1-x_0)n\beta(x_0)\,/\,n = \frac{K}{1-\alpha(1-x_0)}, \tag{S-1}$$

as in the main text (Eqn 1).

The same logic gives the corresponding recursion for the LRE after dispersal, by modifying Eqn (S-1) to:

$$\begin{aligned}\beta_0 &:= K + \alpha\Big(\big(1-d_\mathrm{f}\big)(1-x_1)n\beta_1 + d_\mathrm{f}(1-x)\beta^\circ n\Big)/n \\ &= K + \alpha\Big(\big(1-d_\mathrm{f}\big)(1-x_1)\beta_1 + d_\mathrm{f}(1-x)\beta^\circ\Big) = B\big(\beta_1, x_1\big),\end{aligned} \tag{S-2}$$

which may be interpreted as follows: the first term is a baseline fecundity; the second term overall represents the effect of LRE; the fraction $1-d_\mathrm{f}$ of females derives from the natal patch, in a density $(1-x_1)\beta_1 n$; d_f from other patches, in a density $(1-x)\beta^\circ n$. Assuming that such LRE effect is shared among n adult females, we divide this term by n, obtaining the above expression. We can see that β_0 is a function of $\big(\beta_1, x_1\big)$, and β_1 is a function of $\big(\beta_2, x_2\big)$ (by the same reasoning), and so forth; this means that we need to deal with the nested function of β_0, based upon Lehmann's (2007, 2008) methodology of trans-generational inclusive fitness effects. With this nested structure, we henceforth write:

$$\beta_\tau = B\big(\beta_{\tau+1}, x_{\tau+1}\big) \tag{S-3}$$

for $\tau \geq 0$.

When $x_\tau = x_\bullet = x$ (for any $\tau \geq 0$; neutrality condition), we have

$$\beta^\circ = B\big(\beta^\circ, x\big), \tag{S-4}$$

which immediately gives:

$$\beta^\circ = \frac{K}{1-\alpha(1-x)}, \tag{S-5}$$

whereupon we recover Eqn (3) of the main text.

To guarantee β° be locally stable, we separate the timescales (holding $x_\bullet = x_\tau = x$ for all $\tau \geq 0$) by assuming the sequence of the average fecundity values $\{\widehat{\beta_\tau}\}_{\tau\geq 0}$ (with a hat put to emphasize quasi-equilibrium) is, using Eqn (S-2), determined by:

$$\widehat{\beta_\tau} = K + \alpha(1-x)\Big(\big(1-d_\mathrm{f}\big)\widehat{\beta_{\tau+1}} + d_\mathrm{f}\beta^\circ\Big) = B\Big(\widehat{\beta_{\tau+1}}, x\Big) \tag{S-6}$$

from which we have:

$$\left.\frac{\partial B\big(\widehat{\beta}, x\big)}{\partial\widehat{\beta}}\right|_{\widehat{\beta}=\beta^\circ} = \alpha(1-x)\big(1-d_\mathrm{f}\big), \tag{S-7}$$

which is obviously positive and smaller than 1, thereby guaranteeing the local stability (note, there may be a case in which this quantity is unity but only when $x = d_\mathrm{f} = 0$ and $\alpha = 1$; the necessary condition $x = 0$ is excluded and thereby we focus on the strict inequality).

A-2 Effects of selection

LRE provided from daughters to mothers

Using Eqn (S-1),

$$\left.\frac{\partial \log \beta(x_0)}{\partial x_0}\right|_{x_0=x} = -\alpha\frac{1}{1-\alpha(1-x_0)}, \tag{S-8}$$

and thus:

$$\left.\frac{\partial\beta_0}{\partial x_0}\right|_{x_0=x} = \left.\frac{\partial\beta(x_0)}{\partial x_0}\right|_{x_0=x} = -\alpha\frac{\beta^\circ}{1-\alpha(1-x_0)}. \tag{S-9}$$

LRE provided from offspring to siblings after dispersal

From Eqn (S-2), similar computation gives:

$$\begin{aligned}\left.\frac{\partial\beta_0}{\partial x_1}\right|_{x_\bullet=x_\tau=x} &= \left.\frac{\partial B\big(\beta_1, x_1\big)}{\partial x_1}\right|_{x_\bullet=x_\tau=x} = -\alpha\big(1-d_\mathrm{f}\big)\beta^\circ,\\ \left.\frac{\partial\beta_0}{\partial\beta_1}\right|_{x_\bullet=x_\tau=x} &= \left.\frac{\partial B\big(\beta_1, x_1\big)}{\partial\beta_1}\right|_{x_\bullet=x_\tau=x} = \alpha\big(1-d_\mathrm{f}\big)(1-x).\end{aligned} \tag{S-10}$$

Induction yields:

$$\begin{aligned}\left.\frac{\partial\beta_0}{\partial x_\tau}\right|_{x_\bullet=x_\tau=x} &= \left.\left(\underbrace{\frac{\partial\beta_0}{\partial\beta_1}\cdot\frac{\partial\beta_1}{\partial\beta_2}\cdots\frac{\partial\beta_{\tau-2}}{\partial\beta_{\tau-1}}}_{\tau-1\text{ times}}\cdot\frac{\partial\beta_{\tau-1}}{\partial x_\tau}\right)\right|_{x_\bullet=x_\tau=x} \\ &= \Big(\alpha\big(1-d_\mathrm{f}\big)(1-x)\Big)^{\tau-1}\cdot\Big(-\alpha\big(1-d_\mathrm{f}\big)\beta^\circ\Big) \\ &= -\frac{1}{1-x}\Big(\alpha\big(1-d_\mathrm{f}\big)(1-x)\Big)^{\tau}\cdot\beta^\circ\end{aligned} \tag{S-11}$$

for $\tau \geq 1$. That is, the effect of selection through the expected average of sex ratios in the focal group of the τ-th generation decays geometrically in τ.

B Invasion fitness and the selection gradient

The reproductive success of a focal individual occurs through her daughters (by surviving to obtain the breeding opportunity) and her sons (by obtaining the mating opportunity and mated females' survivorship), and therefore we separate these two terms explicitly by using the class reproductive values (Taylor 1990; Caswell 2001).

B-1 Success via daughters

First of all, the focal female can produce the total number β_0 of eggs. A proportion $1-x_\bullet$ of those eggs develops into female. Therefore the focal individual produce $(1-x_\bullet)\beta_0$ of daughters. We pick one of the daughters;

- with a probability of $1-d_\mathrm{f}$, she stays in her natal patch, subsequently competing with, on average, $\big(1-d_\mathrm{f}\big)(1-x_0)\beta_0$ of juvenile females born in the same patch, plus $d_\mathrm{f}(1-x)\beta^\circ$ of juvenile females each born in a different patch.
- With a probability of d_f, she disperses to an alternative patch to compete with $(1-x)\beta^\circ$ of juvenile females.

Therefore, we have:

$$\begin{aligned}W^\mathbf{f}\big(x_\bullet, x_0, \beta_0\big) &:= \big(1-d_\mathrm{f}\big)\frac{(1-x_\bullet)\beta_0}{\big(1-d_\mathrm{f}\big)(1-x_0)\beta_0 + d_\mathrm{f}(1-x)\beta^\circ} + d_\mathrm{f}\frac{(1-x_\bullet)\beta_0}{(1-x)\beta^\circ} \\ &=: W^\mathbf{f}_\bullet\end{aligned} \tag{S-12}$$

as displayed in Eqn (5) of the main text. At phenotypic neutrality (i.e., $x_\bullet = x_0 = x$ and $\beta_0 = \beta^\circ$), we have $W^\mathbf{f}_\bullet\big(x, x, \beta(x)\big) \equiv 1$ (where, by $\equiv$, we mean "always equal to" or identity).

B-2 Success via sons

The same logic gives success via sons. The focal female produces $x_\bullet\beta_0$ of sons who may compete against on average $\left(1-d_\mathrm{m}\right)x_0\beta_0+d_\mathrm{m}x\beta^\circ$ of juvenile males by staying philopatric (which occurs with a probability of $1-d_\mathrm{m}$); in which case, whether the male gamete survives to the next generation or not now relies on whether the mated female wins a breeding opportunity or not. Because mating is fully random within the patch, $W^\mathbf{f}\left(x_0,x_0,\beta_0\right)$ gives the proportional success of the sons' mating partners. If the sons instead disperse, which occurs with a probability of d_m, then they compete with on average $x\beta^\circ$ of males, and their mating partners' success is $W_\bullet^\mathbf{f}\left(x,x,\beta^\circ\right)\equiv 1$. Therefore,

$$
\begin{aligned}
W_\bullet^\mathbf{m}\left(x_\bullet,x_0,W_0^\mathbf{f},\beta_0\right) &:= \frac{\left(1-d_\mathrm{m}\right)x_\bullet\beta_0}{\left(1-d_\mathrm{m}\right)x_0\beta_0+d_\mathrm{m}x\beta^\circ}\underbrace{\left(\frac{\left(1-d_\mathrm{f}\right)(1-x_0)\beta_0}{\left(1-d_\mathrm{f}\right)(1-x_0)\beta_0+d_\mathrm{f}(1-x)\beta^\circ}+\frac{d_\mathrm{f}(1-x_0)\beta_0}{(1-x)\beta^\circ}\right)}_{=W^\mathbf{f}\left(x_0,x_0,\beta_0\right)=:W_0^\mathbf{f}} \\
&\quad+\frac{d_\mathrm{m}x_\bullet\beta_0}{x\beta^\circ} \\
&=: W_\bullet^\mathbf{m}
\end{aligned}
\tag{S-13}
$$

as displayed in Eqn (6) of the main text.

B-3 Total fitness

The total number of genes of the focal individual transmitted to the next generation is therefore given by $W_\bullet=c_\mathrm{f}W_\bullet^\mathbf{f}+c_\mathrm{m}W_\bullet^\mathbf{m}$ (Taylor 1990; Taylor *et al.* 2007; Gardner *et al.* 2009).

B-4 Evolutionary invasion analyses

We pick the locus Ξ that encodes the sex ratio and write ξ for the genic value of an allele of the locus from a juvenile in the focal patch, with $\xi^\mathbf{f}$ for female and $\xi^\mathbf{m}$ for male, respectively. In addition, we denote the breeding value of (i) the juvenile's mother by $\widetilde{\xi}$, (ii) the average breeding value of the adult females in the same patch in the same generation by η_0, (iii) the average breeding value of all adult females in the metapopulation by ζ, and (iv) the average breeding value of the adult females in the same patch τ-generations prior to the present by η_τ for $\tau\geq 1$.

Under vanishingly small genetic variation in the metapopulation, the direction of selection can be assessed by the sign of:

$$
\frac{\mathrm{d}W_\bullet}{\mathrm{d}\xi}=c_\mathrm{f}\frac{\mathrm{d}W_\bullet^\mathbf{f}}{\mathrm{d}\xi^\mathbf{f}}+c_\mathrm{m}\frac{\mathrm{d}W_\bullet^\mathbf{m}}{\mathrm{d}\xi^\mathbf{f}}, \tag{S-14}
$$

evaluated at $\xi=\xi^\mathbf{f}=\xi^\mathbf{m}=\zeta$, in which c_f (or c_m) is interpreted as the probability that the focal gene lineage is found in a female (or male, respectively). If the above derivative is positive, then selection favours a slightly larger allocation to males.

Further expansions of the partial derivatives are as follows (Lehmann 2007, 2008; Gardner *et al.* 2009):

$$\begin{aligned}\frac{\mathrm{d}W_{\bullet}^{\mathbf{f}}}{\mathrm{d}\xi^{\mathbf{f}}} &= \frac{\partial W_{\bullet}^{\mathbf{f}}}{\partial x_{\bullet}}\cdot\frac{\mathrm{d}x_{\bullet}}{\mathrm{d}\widetilde{\xi}}\cdot\frac{\mathrm{d}\widetilde{\xi}}{\mathrm{d}\xi^{\mathbf{f}}}+\frac{\partial W_{\bullet}^{\mathbf{f}}}{\partial x_0}\cdot\frac{\mathrm{d}x_0}{\mathrm{d}\eta_0}\cdot\frac{\mathrm{d}\eta_0}{\mathrm{d}\xi^{\mathbf{f}}}+\sum_{\tau=0}^{\infty}\frac{\partial W_{\bullet}^{\mathbf{f}}}{\partial\beta_0}\cdot\frac{\partial\beta_0}{\partial x_\tau}\cdot\frac{\mathrm{d}x_\tau}{\mathrm{d}\eta_\tau}\cdot\frac{\mathrm{d}\eta_\tau}{\mathrm{d}\xi^{\mathbf{f}}}\\ &= \frac{\partial W_{\bullet}^{\mathbf{f}}}{\partial x_{\bullet}}Q_{\bullet}^{\mathbf{f}}+\frac{\partial W_{\bullet}^{\mathbf{f}}}{\partial x_0}Q_0^{\mathbf{f}}+\sum_{\tau=0}^{\infty}\frac{\partial W_{\bullet}^{\mathbf{f}}}{\partial\beta_0}\cdot\frac{\partial\beta_0}{\partial x_\tau}Q_\tau^{\mathbf{f}},\end{aligned}\tag{S-15}$$

where, assuming vanishing genetic variation, we have rewritten (i) $\mathrm{d}\widetilde{\xi}/\mathrm{d}\xi^{\mathbf{f}} = Q_{\bullet}^{\mathbf{f}}$ for the consanguinity of the focal female to her own daughters, (ii) $\mathrm{d}\eta_0/\mathrm{d}\xi^{\mathbf{f}} = Q_0^{\mathbf{f}}$ for the consanguinity of the focal female to the juvenile females born to the focal patch in the present generation, (iii) $\mathrm{d}\eta_\tau/\mathrm{d}\xi^{\mathbf{f}} = Q_\tau^{\mathbf{f}}$ for the consanguinity of a random juvenile female and an adult female breeding in the focal patch τ-generations prior to the present (for $\tau \geq 1$), and (iv) we have set the slope of phenotype on genotype to unity: $\mathrm{d}x_{\bullet}/\mathrm{d}\widetilde{\xi} = \mathrm{d}x_0/\mathrm{d}\eta_0 = \mathrm{d}x_\tau/\mathrm{d}\eta_\tau = 1$ (Taylor & Frank 1996; Frank 1998).

The similar expansion yields:

$$\begin{aligned}\frac{\mathrm{d}W_{\bullet}^{\mathbf{m}}}{\mathrm{d}\xi^{\mathbf{m}}} &= \frac{\partial W_{\bullet}^{\mathbf{m}}}{\partial x_{\bullet}}\cdot\frac{\mathrm{d}x_{\bullet}}{\mathrm{d}\widetilde{\xi}}\cdot\frac{\mathrm{d}\widetilde{\xi}}{\mathrm{d}\xi^{\mathbf{m}}}+\frac{\partial W_{\bullet}^{\mathbf{m}}}{\partial x_0}\cdot\frac{\mathrm{d}x_0}{\mathrm{d}\eta_0}\cdot\frac{\mathrm{d}\eta_0}{\mathrm{d}\xi^{\mathbf{m}}}+\sum_{\tau=0}^{\infty}\frac{\partial W_{\bullet}^{\mathbf{m}}}{\partial W_0^{\mathbf{f}}}\cdot\frac{\partial W_0^{\mathbf{f}}}{\partial\beta_0}\cdot\frac{\partial\beta_0}{\partial x_\tau}\cdot\frac{\mathrm{d}x_\tau}{\mathrm{d}\eta_\tau}\cdot\frac{\mathrm{d}\eta_\tau}{\mathrm{d}\xi^{\mathbf{m}}}+\sum_{\tau=0}^{\infty}\frac{\partial W_{\bullet}^{\mathbf{m}}}{\partial\beta_0}\cdot\frac{\partial\beta_0}{\partial x_\tau}\cdot\frac{\mathrm{d}x_\tau}{\mathrm{d}\eta_\tau}\cdot\frac{\mathrm{d}\eta_\tau}{\mathrm{d}\xi^{\mathbf{m}}}\\ &= \frac{\partial W_{\bullet}^{\mathbf{m}}}{\partial x_{\bullet}}Q_{\bullet}^{\mathbf{m}}+\frac{\partial W_{\bullet}^{\mathbf{m}}}{\partial x_0}Q_0^{\mathbf{m}}+\sum_{\tau=0}^{\infty}\frac{\partial W_{\bullet}^{\mathbf{m}}}{\partial W_0^{\mathbf{f}}}\cdot\frac{\partial W_0^{\mathbf{f}}}{\partial\beta_0}\cdot\frac{\partial\beta_0}{\partial x_\tau}Q_\tau^{\mathbf{m}}+\sum_{\tau=0}^{\infty}\frac{\partial W_{\bullet}^{\mathbf{m}}}{\partial\beta_0}\cdot\frac{\partial\beta_0}{\partial x_\tau}Q_\tau^{\mathbf{m}},\end{aligned}\tag{S-16}$$

with basically the same notation as above ($^{\mathbf{f}}$ being replaced with $^{\mathbf{m}}$). Note that the above expression deals with the effects of LRE on the selection gradient as if both types of LRE both occur simultaneously, although we exclusively consider one of the two types of LREs: we use the β_0 of Eqn (S-1) for LRE from daughters to mothers before dispersal, or the β_0 of Eqn (S-2) for LRE from offspring to siblings after dispersal, respectively.

From these, the selection gradient (that separates the LRE effects for $\tau = 0$ and $\tau \geq 1$) reads:

$$\begin{aligned}g(x) := c_{\mathrm{f}}\frac{\mathrm{d}W_{\bullet}^{\mathbf{f}}}{\mathrm{d}\xi^{\mathbf{f}}}+c_{\mathrm{m}}\frac{\mathrm{d}W_{\bullet}^{\mathbf{m}}}{\mathrm{d}\xi^{\mathbf{m}}} &= \underbrace{c_{\mathrm{f}}\left(\frac{\partial W_{\bullet}^{\mathbf{f}}}{\partial x_{\bullet}}Q_{\bullet}^{\mathbf{f}}+\frac{\partial W_{\bullet}^{\mathbf{f}}}{\partial x_0}Q_0^{\mathbf{f}}\right)+c_{\mathrm{m}}\left(\frac{\partial W_{\bullet}^{\mathbf{m}}}{\partial x_{\bullet}}Q_{\bullet}^{\mathbf{m}}+\frac{\partial W_{\bullet}^{\mathbf{m}}}{\partial x_0}Q_0^{\mathbf{m}}+\frac{\partial W_{\bullet}^{\mathbf{m}}}{\partial W_0^{\mathbf{f}}}\frac{\partial W_0^{\mathbf{f}}}{\partial x_0}Q_0^{\mathbf{m}}\right)}_{=:\,g_{\emptyset}(x)}\\ &+\underbrace{\left(c_{\mathrm{f}}\frac{\partial W_{\bullet}^{\mathbf{f}}}{\partial\beta_0}\frac{\partial\beta_0}{\partial x_0}Q_0^{\mathbf{f}}+c_{\mathrm{m}}\frac{\partial W_{\bullet}^{\mathbf{m}}}{\partial\beta_0}\frac{\partial\beta_0}{\partial x_0}Q_0^{\mathbf{m}}+c_{\mathrm{m}}\frac{\partial W_{\bullet}^{\mathbf{m}}}{\partial W_0^{\mathbf{f}}}\frac{\partial W_0^{\mathbf{f}}}{\partial\beta_0}\frac{\partial\beta_0}{\partial x_0}Q_0^{\mathbf{m}}\right)}_{=:\,g_{\tau=0}(x)}\\ &+\underbrace{\sum_{\tau=1}^{\infty}\left(c_{\mathrm{f}}\frac{\partial W_{\bullet}^{\mathbf{f}}}{\partial\beta_0}\frac{\partial\beta_0}{\partial x_\tau}Q_\tau^{\mathbf{f}}+c_{\mathrm{m}}\frac{\partial W_{\bullet}^{\mathbf{m}}}{\partial\beta_0}\frac{\partial\beta_0}{\partial x_\tau}Q_\tau^{\mathbf{m}}+c_{\mathrm{m}}\frac{\partial W_{\bullet}^{\mathbf{m}}}{\partial W_0^{\mathbf{f}}}\frac{\partial W_0^{\mathbf{f}}}{\partial\beta_0}\frac{\partial\beta_0}{\partial x_\tau}Q_\tau^{\mathbf{m}}\right)}_{=:\,g_{\tau\geq 1}(x)},\end{aligned}\tag{S-17}$$

where $g_{\emptyset}(x)$ represents the subcomponent of the selection gradient in the absence of LRE (the first line). Also, $g_{\tau=0}(x)$ and $g_{\tau\geq 1}(x)$, respectively, represent the subcomponent of the total selection gradient for $\tau = 0$ and $\tau \geq$. When we consider the LRE provided from daughters to mothers, or from offspring to siblings after dispersal, we use $g_{\emptyset}(x) + g_{\tau=0}(x)$ (or $g_{\emptyset}(x) + g_{\tau\geq 1}(x)$, respectively) and nullify it to assess the cESS.

B-5 Partial derivatives

Using Eqns (S-12) and (S-13), some algebraic manipulations give the following partial derivatives (each evaluated at neutrality):

$$
\begin{aligned}
\frac{\partial W_{\bullet}^{\mathbf{f}}}{\partial x_{\bullet}} &= -\frac{1}{1-x}; \\
\frac{\partial W_{\bullet}^{\mathbf{f}}}{\partial x_0} &= \frac{\left(1-d_{\mathrm{f}}\right)^2}{1-x} = \frac{\sigma_{\mathrm{RC}}}{1-x}; \\
\frac{\partial W_{\bullet}^{\mathbf{m}}}{\partial x_{\bullet}} &= \frac{1}{x}; \\
\frac{\partial W_{\bullet}^{\mathbf{m}}}{\partial x_0} &= \frac{-\left(1-d_{\mathrm{m}}\right)^2}{x} = -\frac{\sigma_{\mathrm{MC}}}{x}; \\
\frac{\partial W_{\bullet}^{\mathbf{f}}}{\partial \beta_0} &= \frac{1-\left(1-d_{\mathrm{f}}\right)^2}{\beta^{\circ}} = \frac{1-\sigma_{\mathrm{RC}}}{\beta^{\circ}}; \\
\frac{\partial W_{\bullet}^{\mathbf{m}}}{\partial W_0^{\mathbf{f}}} \cdot \frac{\partial W_0^{\mathbf{f}}}{\partial \beta_0} &= \left(1-d_{\mathrm{m}}\right) \cdot \frac{1-\left(1-d_{\mathrm{f}}\right)^2}{\beta^{\circ}} = \frac{\sigma_{\mathrm{MB}}}{\beta^{\circ}}; \\
\frac{\partial W_0^{\mathbf{f}}}{\partial x_0} &= -\frac{1-\left(1-d_{\mathrm{f}}\right)^2}{1-x} = -\frac{1-\sigma_{\mathrm{RC}}}{1-x}; \\
\frac{\partial W_{\bullet}^{\mathbf{m}}}{\partial \beta_0} &= \frac{1-\left(1-d_{\mathrm{m}}\right)^2}{\beta^{\circ}} = \frac{1-\sigma_{\mathrm{MC}}}{\beta^{\circ}},
\end{aligned}
\tag{S-18}
$$

in which we have written (i) $\left(1-d_{\mathrm{f}}\right)^2 = \sigma_{\mathrm{RC}}$ for the strength of local resource competition among females, (ii) $\left(1-d_{\mathrm{m}}\right)^2 = \sigma_{\mathrm{MC}}$ for the strength of local mate competition among males, and (iii) $\sigma_{\mathrm{MB}} = \left(1-d_{\mathrm{m}}\right)\left(1-\left(1-d_{\mathrm{f}}\right)^2\right)$ for the degree of local mating bonus. Mathematically, these are: (i) the probability that a juvenile female competes for resource with another juvenile female born in the same patch, (ii) the probability that a juvenile male competes for mating opportunity with another juvenile male born in the same patch, and (iii) the joint probability that a male mate with a juvenile female born in the same patch and also she does not competite with any of juvenile females born in the same patch.

B-6 Hamilton's rule

If we divide both sides of Eqn (S-17) by the consanguinity coefficient of an adult female to herself, $Q_\bullet (> 0)$, we get:

$$\begin{aligned}\frac{g(x)}{Q_\bullet} &= \underbrace{c_\mathrm{f}\left(\frac{\partial W^\mathbf{f}_\bullet}{\partial x_\bullet} R^\mathbf{f}_\bullet + \frac{\partial W^\mathbf{f}_\bullet}{\partial x_0} R^\mathbf{f}_0\right) + c_\mathrm{m}\left(\frac{\partial W^\mathbf{m}_\bullet}{\partial x_\bullet} R^\mathbf{m}_\bullet + \frac{\partial W^\mathbf{m}_\bullet}{\partial x_0} R^\mathbf{m}_0 + \frac{\partial W^\mathbf{m}_\bullet}{\partial W^\mathbf{f}_0}\frac{\partial W^\mathbf{f}_0}{\partial x_0} R^\mathbf{m}_0\right)}_{\text{no LRE}} \\ &\quad + \underbrace{\sum_{\tau=0}^{\infty}\left(c_\mathrm{f}\frac{\partial W^\mathbf{f}_\bullet}{\partial \beta_0}\frac{\partial \beta_0}{\partial x_\tau} R^\mathbf{f}_\tau + c_\mathrm{m}\frac{\partial W^\mathbf{m}_\bullet}{\partial \beta_0}\frac{\partial \beta_0}{\partial x_\tau} R^\mathbf{m}_\tau + c_\mathrm{m}\frac{\partial W^\mathbf{m}_\bullet}{\partial W^\mathbf{f}_0}\frac{\partial W^\mathbf{f}_0}{\partial \beta_0}\frac{\partial \beta_0}{\partial x_\tau} R^\mathbf{m}_\tau\right)}_{\text{LRE effects (intra: } \tau = 0\text{; trans: } \tau \geq 1)};\end{aligned} \tag{S-19}$$

Inserting Eqn (S-18) into Eqn (S-19) without making explicit the relatedness coefficients (as functions of dispersal rates and n), we get:

$$\begin{aligned}\frac{g(x)}{Q_\bullet} &= c_\mathrm{f}\left(\frac{-1}{1-x} R^\mathbf{f}_\bullet + \frac{\sigma_\mathrm{RC}}{1-x} R^\mathbf{f}_0\right) + c_\mathrm{m}\left(\frac{1}{x} R^\mathbf{m}_\bullet - \frac{\sigma_\mathrm{MC}}{x} R^\mathbf{m}_0 - \frac{\sigma_\mathrm{MB}}{1-x} R^\mathbf{m}_0\right) \\ &\quad + \sum_{\tau=0}^{\infty}\frac{\partial \beta_0}{\partial x_\tau}\left(c_\mathrm{f}\frac{1-\sigma_\mathrm{RC}}{\beta^\circ} R^\mathbf{f}_\tau + c_\mathrm{m}\frac{1-\sigma_\mathrm{MC}}{\beta^\circ} R^\mathbf{m}_\tau + c_\mathrm{m}\frac{\sigma_\mathrm{MB}}{\beta^\circ} R^\mathbf{m}_\tau\right).\end{aligned} \tag{S-20}$$

We note that Hamilton's regression coefficient of relatedness is represented by a fractional form, of (i) the consanguinity of a focal class of individuals (typically one of the adult females in a given patch) to some classes of individuals, divided by (ii) the consanguinity of the focal actor to itself ($Q_\bullet$; Michod & Hamilton 1980; Taylor 1988; Bulmer 1994).

B-7 No LRE

When $\alpha = 0$ (no LRE), the second line of Eqn (S-20) is null, and thus Eqn (S-20) vanishes if:

$$\frac{g(x)}{Q_\bullet} = c_\mathrm{f}\left(\frac{-1}{1-x} R^\mathbf{f}_\bullet + \frac{\sigma_\mathrm{RC}}{1-x} R^\mathbf{f}_0\right) + c_\mathrm{m}\left(\frac{1}{x} R^\mathbf{m}_\bullet - \frac{\sigma_\mathrm{MC}}{x} R^\mathbf{m}_0 - \frac{\sigma_\mathrm{MB}}{1-x} R^\mathbf{m}_0\right) = 0, \tag{S-21}$$

which is equivalent to:

$$\frac{g(x)}{Q_\bullet} = \frac{1}{x(1-x)}\left(c_\mathrm{f}\left(-xR^\mathbf{f}_\bullet + \sigma_\mathrm{RC} x R^\mathbf{f}_0\right) + c_\mathrm{m}\left((1-x)R^\mathbf{m}_\bullet - (1-x)\sigma_\mathrm{MC} R^\mathbf{m}_0 - x\sigma_\mathrm{MB} R^\mathbf{m}_0\right)\right) = 0. \tag{S-22}$$

Multiplying $x(1-x)>0$ yields:

$$
\begin{aligned}
&x(1-x)\frac{g(x)}{Q_\bullet} \\
&= c_\mathrm{m}\Big(R^{\mathbf{m}}_\bullet - \sigma_{\mathrm{MC}} R^{\mathbf{m}}_0\Big) + c_\mathrm{f}\Big(-R^{\mathbf{f}}_\bullet + \sigma_{\mathrm{RC}} R^{\mathbf{f}}_0\Big)x + c_\mathrm{m}\Big(-R^{\mathbf{m}}_\bullet + \sigma_{\mathrm{MC}} R^{\mathbf{m}}_0 - \sigma_{\mathrm{MB}} R^{\mathbf{m}}_0\Big)x \\
&= \underbrace{\Big(c_\mathrm{m}\Big(R^{\mathbf{m}}_\bullet - (\sigma_{\mathrm{MC}}-\sigma_{\mathrm{MB}})R^{\mathbf{m}}_0\Big) + c_\mathrm{f}\Big(R^{\mathbf{f}}_\bullet - \sigma_{\mathrm{RC}} R^{\mathbf{f}}_0\Big)\Big)}_{>0} \times \left(\underbrace{\frac{c_\mathrm{m}\Big(R^{\mathbf{m}}_\bullet - \sigma_{\mathrm{MC}} R^{\mathbf{m}}_0\Big)}{c_\mathrm{m}\Big(R^{\mathbf{m}}_\bullet - (\sigma_{\mathrm{MC}}-\sigma_{\mathrm{MB}})R^{\mathbf{m}}_0\Big) + c_\mathrm{f}\Big(R^{\mathbf{f}}_\bullet - \sigma_{\mathrm{RC}} R^{\mathbf{f}}_0\Big)}}_{=:\widehat{x_\emptyset}} - x \right) \\
&= 0,
\end{aligned}
\tag{S-23}
$$

which obtains the expression for cESS (candidate ESS), of

$$
\widehat{x_\emptyset} = \frac{c_\mathrm{m}\Big(R^{\mathbf{m}}_\bullet - \sigma_{\mathrm{MC}} R^{\mathbf{m}}_0\Big)}{c_\mathrm{m}\Big(R^{\mathbf{m}}_\bullet - (\sigma_{\mathrm{MC}}-\sigma_{\mathrm{MB}})R^{\mathbf{m}}_0\Big) + c_\mathrm{f}\Big(R^{\mathbf{f}}_\bullet - \sigma_{\mathrm{RC}} R^{\mathbf{f}}_0\Big)}, \tag{S-24}
$$

which recovers the cESS displayed in Eqns (8) and (9) of the main text. By making the relatedness coefficients explicit with $d_\mathrm{f}, d_\mathrm{m}$ and n, we can obtain the numerical value for $\widehat{x_\emptyset}$ to generate Fig 3 of the main text.

From this manipulation from Eqns (S-21) to (S-23) we can see that it is of great use to scale $g(x)$ by:

$$
\begin{aligned}
&g(x)>0 \\
\iff &\underbrace{\frac{x(1-x)g(x)}{Q_\bullet\Big(c_\mathrm{m}\Big(R^{\mathbf{m}}_\bullet - (\sigma_{\mathrm{MC}}-\sigma_{\mathrm{MB}})R^{\mathbf{m}}_0\Big) + c_\mathrm{f}\Big(R^{\mathbf{f}}_\bullet - \sigma_{\mathrm{RC}} R^{\mathbf{f}}_0\Big)\Big)}}_{=\widehat{x_\emptyset}-x,\ \text{when}\ \alpha=0} > 0,
\end{aligned}
\tag{S-25}
$$

as it gives a simple measure of the direction of selection (see the next paragraph). We therefore define:

$$
H(x) := \frac{x(1-x)}{Q_\bullet\Big(c_\mathrm{m}\Big(R^{\mathbf{m}}_\bullet - (\sigma_{\mathrm{MC}}-\sigma_{\mathrm{MB}})R^{\mathbf{m}}_0\Big) + c_\mathrm{f}\Big(R^{\mathbf{f}}_\bullet - \sigma_{\mathrm{RC}} R^{\mathbf{f}}_0\Big)\Big)} \cdot g(x) \tag{S-26}
$$

and refer to this as 'scaled Hamilton's rule,' presented in Results of the main text (Eqns 9 – 12).

One can see why this rescaling is useful, by plugging $\alpha = 0$ into the scaled Hamilton's rule to recover the last term in Eqn (S-23): $H(x)|_{\alpha=0} = \widehat{x_\emptyset} - x$. On the other hand, with $\alpha > 0$, $H(x) = \widehat{x_\emptyset} - x +$ (Sum of LRE terms as a function of x). That is, this transformation explicitly separates the classic Hamiltonian sex ratio as a linear term, and simply by inspecting the last term we are able to predict the LRE-driven selective forces.

B-8 Scaled Hamilton's rule for the LRE from daughters to mothers before dispersal

Substituting Eqns (S-10) and (S-11) into Eqn (S-20) and rescaling it to scaled Hamilton's rule $H(x)$ defined in Eqn (S-26) (but without making the relatedness coefficients explicit (while extracting the $\tau = 0$ term from $\sum_{\tau \geq 0}$) in Eqn (S-20)), we arrive at:

$$H(x) = \widehat{x_\emptyset} - x - x\frac{\alpha(1-x)}{1-\alpha(1-x)} \cdot \underbrace{\frac{c_\mathrm{f} R_0^\mathbf{f}(1-\sigma_\mathrm{RC}) + c_\mathrm{m} R_0^\mathbf{m}(1-(\sigma_\mathrm{MC}-\sigma_\mathrm{MB}))}{c_\mathrm{f}\left(R_\bullet^\mathbf{f} - \sigma_\mathrm{RC} R_0^\mathbf{f}\right) + c_\mathrm{m}\left(R_\bullet^\mathbf{m} - (\sigma_\mathrm{MC}-\sigma_\mathrm{MB}) R_0^\mathbf{m}\right)}}_{=:\kappa} \tag{S-27}$$

and define κ as above.

B-9 Scaled Hamilton's rule for the LRE from offspring to siblings after dispersal

Similar computation yields:

$$\begin{aligned} H(x) &= \widehat{x_\emptyset} - x + x(1-x)\sum_{\tau=1}^{+\infty} \frac{\left(c_\mathrm{f}(1-\sigma_\mathrm{RC}) R_\tau^\mathbf{f} + c_\mathrm{m}(1-\sigma_\mathrm{MC}+\sigma_\mathrm{MB}) R_\tau^\mathbf{m}\right)}{c_\mathrm{f}\left(R_\bullet^\mathbf{f} - \sigma_\mathrm{RC} R_0^\mathbf{f}\right) + c_\mathrm{m}\left(R_\bullet^\mathbf{m} - (\sigma_\mathrm{MC}-\sigma_\mathrm{MB}) R_0^\mathbf{m}\right)} \cdot \frac{1}{\beta^\circ} \cdot \overbrace{\frac{\partial \beta_0}{\partial x_\tau}}^{\text{Eqn (S-11)}} \\ &= \widehat{x_\emptyset} - x - x\sum_{\tau=1}^{+\infty} \frac{c_\mathrm{f}(1-\sigma_\mathrm{RC}) R_\tau^\mathbf{f} + c_\mathrm{m}(1-\sigma_\mathrm{MC}+\sigma_\mathrm{MB}) R_\tau^\mathbf{m}}{c_\mathrm{f}\left(R_\bullet^\mathbf{f} - \sigma_\mathrm{RC} R_0^\mathbf{f}\right) + c_\mathrm{m}\left(R_\bullet^\mathbf{m} - (\sigma_\mathrm{MC}-\sigma_\mathrm{MB}) R_0^\mathbf{m}\right)} \left(\alpha(1-x)\left(1-d_\mathrm{f}\right)\right)^\tau \end{aligned} \tag{S-28}$$

(Lehmann 2007, 2008). So we must obtain recursions for $R_\tau^\mathbf{f}, R_\tau^\mathbf{m}$ (below).

B-10 Consanguinities within the same generation

We denote the consanguinity between:

(i) a juvenile female and male sharing the same patch by $Q_0^\mathbf{fm}$,

(ii) two juvenile males sharing the same patch by $Q_0^\mathbf{mm}$, and

(iii) two juvenile females sharing the same patch by $Q_0^\mathbf{ff}$,

each immediately after birth (and thus before male dispersal) in the same generation $\tau = 0$.

Using the standard coalescent argument for haplodioloids, we get:

$$\begin{aligned} Q_0^\mathbf{ff} &= \frac{1}{n}\left(\frac{Q_\bullet + 2\left(1-d_\mathrm{m}\right) Q_0^\mathbf{fm} + 1}{4}\right) + \frac{n-1}{n}\sigma_\mathrm{RC}\left(\frac{Q_0^\mathbf{ff} + 2\left(1-d_\mathrm{m}\right) Q_0^\mathbf{fm} + \left(1-d_\mathrm{m}\right)^2 Q_0^\mathbf{mm}}{4}\right), \\ Q_0^\mathbf{fm} &= \frac{1}{n}\left(\frac{Q_\bullet + \left(1-d_\mathrm{m}\right) Q_0^\mathbf{fm}}{2}\right) + \frac{n-1}{n}\sigma_\mathrm{RC}\left(\frac{Q_0^\mathbf{ff} + \left(1-d_\mathrm{m}\right) Q_0^\mathbf{fm}}{2}\right), \\ Q_0^\mathbf{mm} &= \frac{1}{n} Q_\bullet + \frac{n-1}{n}\sigma_\mathrm{RC} Q_0^\mathbf{ff} \end{aligned} \tag{S-29}$$

(Taylor 1988; Johnstone *et al.* 2012), which are affine equations (and thus have unique solutions), and therefore

we can determine each consanguinity coefficient.

The consanguinity of an adult female to

(i) herself is $Q_\bullet = \left(1 + \left(1 - d_\mathrm{m}\right) Q_0^{\mathbf{fm}}\right)/2$,

(ii) her daughter is $Q_\bullet^{\mathbf{f}} := \left(Q_\bullet + \left(1 - d_\mathrm{m}\right) Q_0^{\mathbf{fm}}\right)/2$,

(iii) her son is $Q_\bullet^{\mathbf{m}} := \left(1 + \left(1 - d_\mathrm{m}\right) Q_0^{\mathbf{fm}}\right)/2$,

(iv) a random juvenile female born in the same patch in the present generation is $Q_0^{\mathbf{f}} := \left(Q_\bullet^{\mathbf{f}} + \left(1 - d_\mathrm{m}\right) Q_0^{\mathbf{fm}}\right)/n + \left(1 - d_\mathrm{f}\right)^2 (1 - 1/n) \left(Q_0^{\mathbf{ff}} + \left(1 - d_\mathrm{m}\right) Q_0^{\mathbf{fm}}\right)/2$, which turns out to equal $Q_0^{\mathbf{fm}}$, and

(v) a random juvenile male in the same patch born in the present generation is $Q_0^{\mathbf{m}} := Q_\bullet^{\mathbf{m}}/n + \left(1 - d_\mathrm{f}\right)^2 (1 - 1/n) Q_0^{\mathbf{ff}} = Q_0^{\mathbf{mm}}$.

Also, the relatedness coefficient, from the adult female's perspective (Taylor 1988; Bulmer 1994), to

(i) her daughter is $R_\bullet^{\mathbf{f}} := Q_\bullet^{\mathbf{f}}/Q_\bullet$,

(ii) a random juvenile female on the same patch born in the present generation is $R_0^{\mathbf{f}} := Q_0^{\mathbf{f}}/Q_\bullet$,

(iii) her son is $R_\bullet^{\mathbf{m}} := Q_\bullet^{\mathbf{m}}/Q_\bullet$, and

(iv) a random juvenile male on the same patch born in the present generation is $R_0^{\mathbf{m}} := Q_0^{\mathbf{m}}/Q_\bullet$.

Proof for $\kappa = 1/n$ *for* $d_m = 0$

We here show that $d_\mathrm{m} = 0$ gives $\kappa = 1/n$ as shown in Eqn (11) by using Taylor's (1992) arguments.

First, plugging $d_\mathrm{m} = 0$ into Eqn (S-29), we get:

$$Q_0^{\mathbf{ff}} = \frac{1}{n}\left(\frac{Q_\bullet + 2Q_0^{\mathbf{fm}} + 1}{4}\right) + \frac{n-1}{n}\sigma_{\mathrm{RC}}\left(\frac{Q_0^{\mathbf{ff}} + 2Q_0^{\mathbf{fm}} + Q_0^{\mathbf{mm}}}{4}\right), \tag{S-30}$$

$$Q_0^{\mathbf{fm}} = \frac{1}{n}\left(\frac{Q_\bullet + Q_0^{\mathbf{fm}}}{2}\right) + \frac{n-1}{n}\sigma_{\mathrm{RC}}\left(\frac{Q_0^{\mathbf{ff}} + Q_0^{\mathbf{fm}}}{2}\right), \tag{S-31}$$

$$Q_0^{\mathbf{mm}} = \frac{1}{n}Q_\bullet + \frac{n-1}{n}\sigma_{\mathrm{RC}} Q_0^{\mathbf{ff}}, \tag{S-32}$$

with:

$$\begin{aligned} Q_\bullet &= \frac{1 + Q_0^{\mathbf{fm}}}{2}, \\ Q_0^{\mathbf{f}} &= Q_0^{\mathbf{fm}}, \\ Q_0^{\mathbf{m}} &= Q_0^{\mathbf{mm}}, \\ Q_\bullet^{\mathbf{f}} &= \frac{Q_\bullet + Q_0^{\mathbf{fm}}}{2}, \\ Q_\bullet^{\mathbf{m}} &= \frac{1 + Q_0^{\mathbf{fm}}}{2}. \end{aligned} \tag{S-33}$$

Second, computing $Q_0^{\mathbf{ff}} - \left(Q_0^{\mathbf{fm}} + Q_0^{\mathbf{mm}}\right)/2$ from Eqns (S-30) to (S-32) (separating LHS and RHS, while using Eqn (S-33)) yields:

$$Q_0^{\mathbf{ff}} - \frac{Q_0^{\mathbf{fm}} + Q_0^{\mathbf{mm}}}{2} = -\sigma_{\mathrm{RC}}\frac{n-1}{n}\left(Q_0^{\mathbf{ff}} - \frac{Q_0^{\mathbf{fm}} + Q_0^{\mathbf{mm}}}{2}\right), \tag{S-34}$$

thus implying

$$Q_0^{\mathbf{ff}} = \frac{Q_0^{\mathbf{fm}} + Q_0^{\mathbf{mm}}}{2} \tag{S-35}$$

at equilibrium.

Third, let us repeat the definition of κ (for arbitrary d_{m}):

$$\kappa = \frac{c_{\mathrm{f}}R_0^{\mathbf{f}}(1-\sigma_{\mathrm{RC}}) + c_{\mathrm{m}}R_0^{\mathbf{m}}(1-(\sigma_{\mathrm{MC}}-\sigma_{\mathrm{MB}}))}{c_{\mathrm{f}}\left(R_\bullet^{\mathbf{f}} - \sigma_{\mathrm{RC}}R_0^{\mathbf{f}}\right) + c_{\mathrm{m}}\left(R_\bullet^{\mathbf{m}} - (\sigma_{\mathrm{MC}}-\sigma_{\mathrm{MB}})R_0^{\mathbf{m}}\right)}, \tag{S-36}$$

into which we substitute $d_{\mathrm{m}} = 0$ to get:

$$\begin{aligned}\kappa\Big|_{d_{\mathrm{m}}=0} &= \frac{c_{\mathrm{f}}R_0^{\mathbf{f}}(1-\sigma_{\mathrm{RC}}) + c_{\mathrm{m}}R_0^{\mathbf{m}}(1-\sigma_{\mathrm{RC}})}{c_{\mathrm{f}}\left(R_\bullet^{\mathbf{f}} - \sigma_{\mathrm{RC}}R_0^{\mathbf{f}}\right) + c_{\mathrm{m}}\left(R_\bullet^{\mathbf{m}} - \sigma_{\mathrm{RC}}R_0^{\mathbf{m}}\right)} \\ &= \frac{c_{\mathrm{f}}Q_0^{\mathbf{f}}(1-\sigma_{\mathrm{RC}}) + c_{\mathrm{m}}Q_0^{\mathbf{m}}(1-\sigma_{\mathrm{RC}})}{c_{\mathrm{f}}\left(Q_\bullet^{\mathbf{f}} - \sigma_{\mathrm{RC}}Q_0^{\mathbf{f}}\right) + c_{\mathrm{m}}\left(Q_\bullet^{\mathbf{m}} - \sigma_{\mathrm{RC}}Q_0^{\mathbf{m}}\right)}\end{aligned} \tag{S-37}$$

(where we used $\sigma_{\mathrm{MC}} - \sigma_{\mathrm{MB}} \equiv \sigma_{\mathrm{RC}}$ when $d_{\mathrm{m}} = 0$). If we, for now, define $\rho = \left(c_{\mathrm{f}}Q_0^{\mathbf{f}} + c_{\mathrm{m}}Q_0^{\mathbf{m}}\right)/\left(c_{\mathrm{f}}Q_\bullet^{\mathbf{f}} + c_{\mathrm{m}}Q_\bullet^{\mathbf{m}}\right)$, then:

$$\kappa\Big|_{d_{\mathrm{m}}=0} = \frac{\rho(1-\sigma_{\mathrm{RC}})}{1-\rho\sigma_{\mathrm{RC}}}. \tag{S-38}$$

$c_{\mathrm{f}}\times$Eqn (S-31) plus $c_{\mathrm{m}}\times$Eqn (S-32) gives:

$$\begin{aligned}&c_{\mathrm{f}}\overbrace{Q_0^{\mathbf{fm}}}^{=Q_0^{\mathbf{f}};\ \text{Eqn (S-33)}} + c_{\mathrm{m}}\overbrace{Q_0^{\mathbf{mm}}}^{=Q_0^{\mathbf{m}};\ \text{Eqn (S-33)}} = \frac{1}{n}\left(c_{\mathrm{f}}\overbrace{\frac{Q_\bullet + Q_0^{\mathbf{fm}}}{2}}^{=Q_\bullet^{\mathbf{f}};\ \text{Eqn (S-33)}} + c_{\mathrm{m}}\overbrace{Q_\bullet}^{=Q_\bullet^{\mathbf{m}};\ \text{Eqn (S-33)}}\right) + \frac{n-1}{n}\sigma_{\mathrm{RC}}\underbrace{\left(c_{\mathrm{f}}\frac{Q_0^{\mathbf{ff}} + Q_0^{\mathbf{fm}}}{2} + c_{\mathrm{m}}Q_0^{\mathbf{ff}}\right)}_{=(\star)} \\ &\iff c_{\mathrm{f}}Q_0^{\mathbf{f}} + c_{\mathrm{m}}Q_0^{\mathbf{m}} = \frac{1}{n}\left(c_{\mathrm{f}}Q_\bullet^{\mathbf{f}} + c_{\mathrm{m}}Q_\bullet^{\mathbf{m}}\right) + \frac{n-1}{n}\sigma_{\mathrm{RC}}\underbrace{\left(c_{\mathrm{f}}Q_0^{\mathbf{f}} + c_{\mathrm{m}}Q_0^{\mathbf{m}}\right)}_{=(\star)}\end{aligned} \tag{S-39}$$

(where $(\star)$s are, after algebraic manipulations, found to equal for both diploids and haplodiploids), which,

dividing both sides by $\left(c_{\mathrm{f}}Q^{\mathbf{f}}_{\bullet}+c_{\mathrm{m}}Q^{\mathbf{m}}_{\bullet}\right)$ and then using ρ, implies:

$$\rho=\frac{1}{n}+\frac{n-1}{n}\sigma_{\mathrm{RC}}\rho, \tag{S-40}$$

which by solving for $1/n$ gives:

$$\frac{1}{n}=\frac{\rho(1-\sigma_{\mathrm{RC}})}{1-\rho\sigma_{\mathrm{RC}}}, \tag{S-41}$$

which is equal to Eqn (S-38). Hence we conclude that $\kappa=1/n$ when $d_{\mathrm{m}}=0$ (Eqn 11 of the main text).

B-11 Consanguinities across generations in the same patch

We focus on haplodiploid genetics, and designate $Q^{\mathbf{f}}_{\tau}$ (or $Q^{\mathbf{m}}_{\tau}$) for the probability of consanguinity between (i) a juvenile female (or male) randomly sampled right after its birth in the present generation in a patch and (ii) a random adult female in the same patch in the τ-th generation ($\tau\geq 0$). To develop the recursion between $\mathbf{q}_{\tau+1}=\left(Q^{\mathbf{f}}_{\tau+1},Q^{\mathbf{m}}_{\tau+1}\right)^{\top}$ and $\mathbf{q}_{\tau}=\left(Q^{\mathbf{f}}_{\tau},Q^{\mathbf{m}}_{\tau}\right)^{\top}$ (with $^{\top}$ for transpose), we consider the following, three classes of individuals living in the same patch but potentially in different time epochs:

(i) adult females in the $\tau+1$-st generation (representatively named $\mathcal{A}_{\tau+1}$),

(ii) juvenile females or males born from the adult females living in the generation $\tau=1$ (named $\mathcal{J}^{\mathbf{f}}_{1}$ for female and $\mathcal{J}^{\mathbf{m}}_{1}$ for male), and

(iii) juvenile females or males born from the adult females in the current generation $\tau=0$ (named $\mathcal{J}^{\mathbf{f}}_{0}$ for female and $\mathcal{J}^{\mathbf{m}}_{0}$ for male).

The consanguinity between (i) and (ii) is by definition $\mathbf{q}_{\tau}$ and that between (i) and (iii) is $\mathbf{q}_{\tau+1}$. The initial condition ($\tau=0$) reads $Q^{\mathbf{f}}_{0}=Q^{\mathbf{fm}}_{0}$ (which is the consanguinity of a random juvenile female born in $\tau=0$ and a random adult reproducing in the same generation) and $Q^{\mathbf{m}}_{0}=Q^{\mathbf{mm}}_{0}$ (which is the consanguinity of a random juvenile male born in $\tau=0$ and a random adult reproducing in the same generation). We depict a conceptual illustration in SI Fig 2, in which both actors' and recipients' perspectives (or inclusive-fitness and neighbor-modulated fitness approaches) agree, as in Taylor *et al.* (2007). Below, we write $\mathcal{A}_{\tau}$ for a random adult female sampled from a patch in the τ-th generation, and $\mathcal{J}^{\mathbf{f}}_{0}$ (or $\mathcal{J}^{\mathbf{m}}_{0}$) for a random juvenile female (or male) born from an adult female in the same patch in the present generation (respectively). The key to the calculation for the coefficients of consanguinity across generations is to compute the consanguinity between $\left(\mathcal{J}^{\mathbf{f}}_{0},\mathcal{J}^{\mathbf{m}}_{0}\right)$ and $\left(\mathcal{J}^{\mathbf{f}}_{1},\mathcal{J}^{\mathbf{m}}_{1}\right)$, not between $\mathcal{A}_{\tau}$ and $\mathcal{A}_{\tau+1}$ (SI Fig 2), as the latter may be trickier (despite being equivalent).

Recursion for $Q^{\mathbf{f}}_{\tau+1}$

First we consider the probability that $\mathcal{A}_{\tau+1}$ (a random adult female sampled from a patch in the $\tau+1$-st generation) and $\mathcal{J}^{\mathbf{f}}_{0}$ (a random juvenile female born from an adult female in the same patch in the present generation) share an allele of identity-by-descent (IBD). For this to occur, it entails that $\mathcal{J}^{\mathbf{f}}_{0}$'s mother be of philopatric origin ($1-d_{\mathrm{f}}$); given this, $\mathcal{J}^{\mathbf{f}}_{0}$'s allele derives maternally with a probability of $1/2$, in which case IBD probability is $Q^{\mathbf{f}}_{\tau}$; otherwise, it derives from a father ($1/2$) born in the same patch ($1-d_{\mathrm{m}}$), in which case IBD

probability is $Q^{\mathbf{m}}_{\tau}$. Hence,

$$Q^{\mathbf{f}}_{\tau+1} = \frac{1}{2}\left(1-d_{\mathrm{f}}\right)Q^{\mathbf{f}}_{\tau} + \frac{1}{2}\left(1-d_{\mathrm{f}}\right)\left(1-d_{\mathrm{m}}\right)Q^{\mathbf{m}}_{\tau}. \tag{S-42}$$

Recursion for $Q^{\mathbf{m}}_{\tau+1}$

Similarly, we consider the probability that $\mathcal{A}_{\tau+1}$ (a random adult female sampled from a patch in the $\tau+1$-st generation) and $\mathcal{J}^{\mathbf{m}}_{0}$ (a random juvenile male born to the adult females in the same patch in the present generation) share an allele of IBD. Given that $\mathcal{J}^{\mathbf{m}}_{0}$'s mother be of philopatric origin (which occurs with a probability of $1-d_{\mathrm{f}}$), $\mathcal{J}^{\mathbf{m}}_{0}$'s allele derives certainly maternally (probability 1), in which case IBD probability is $Q^{\mathbf{f}}_{\tau}$, yielding:

$$Q^{\mathbf{m}}_{\tau+1} = \left(1-d_{\mathrm{f}}\right)Q^{\mathbf{f}}_{\tau}. \tag{S-43}$$

Vector form of the recursions

In a vector form, Eqn (S-42) and Eqn (S-43), divided by $Q_{\bullet}$ read:

$$\begin{pmatrix} R^{\mathbf{f}}_{\tau+1} \\ R^{\mathbf{m}}_{\tau+1} \end{pmatrix} = \underbrace{\begin{pmatrix} 1-d_{\mathrm{f}} & 0 \\ 0 & 1-d_{\mathrm{f}} \end{pmatrix}}_{\text{female dispersal}} \underbrace{\begin{pmatrix} \pi_{\mathrm{ff}} & \pi_{\mathrm{fm}} \\ \pi_{\mathrm{mf}} & \pi_{\mathrm{mm}} \end{pmatrix}}_{\text{inheritance}} \underbrace{\begin{pmatrix} 1 & 0 \\ 0 & 1-d_{\mathrm{m}} \end{pmatrix}}_{\text{male dispersal}} \begin{pmatrix} R^{\mathbf{f}}_{\tau} \\ R^{\mathbf{m}}_{\tau} \end{pmatrix} \tag{S-44}$$

$$= \underbrace{\begin{pmatrix} \frac{1-d_{\mathrm{f}}}{2} & \frac{(1-d_{\mathrm{f}})(1-d_{\mathrm{m}})}{2} \\ 1-d_{\mathrm{f}} & 0 \end{pmatrix}}_{=\mathbf{P}} \begin{pmatrix} R^{\mathbf{f}}_{\tau} \\ R^{\mathbf{m}}_{\tau} \end{pmatrix}, \tag{S-45}$$

where π_{XY} represents the probability that a juvenile of sex X derives its gene from an adult of sex Y, and we have substituted $\pi_{\mathrm{ff}} = \pi_{\mathrm{fm}} = 1/2, \pi_{\mathrm{mf}} = 1$ and $\pi_{\mathrm{mm}} = 0$ for haplodiploids. Note that in haploids or diploids, πs are all 1/2, and $R^{\mathbf{f}}_{\tau} = R^{\mathbf{m}}_{\tau} = \left(1-d_{\mathrm{f}}\right)^{\tau}\left(\left(2-d_{\mathrm{m}}\right)/2\right)^{\tau} R^{\mathbf{f}}_{0}$.

In Eqn (S-44), the first matrix determines the decay of genetic relatedness due to female emigration, in which mated females disperse and therefore mated males' gametes may emigrate together (the bottom-right element should not be 1); the second describes the genetic inheritance, or movement of genes between sexes due to mating; the third represents the male dispersal, by which juvenile females' gametes do not disperse. This decomposition is useful when one wishes to expand the model to different order of lifecycle events (e.g., DDM model in Wild & Taylor 2004 in which female and male both disperse prior to mating).

Hamilton's rule

Now let $\mathbf{V} := \alpha\left(1-d_{\mathrm{f}}\right)(1-x)\mathbf{P}$ (see Eqn (S-28)); then its spectral radius is less than unity in modulus and therefore $(\mathbf{I}-\mathbf{V})^{-1}\mathbf{V} = \sum_{\tau=1}^{\infty}\mathbf{V}^{\tau}$ does exist (with $\mathbf{I}$ the identity). Then, Eqn (S-28) becomes:

$$\begin{aligned} H(x) &= \widehat{x_{\emptyset}} - x - x\left(c_{\mathrm{f}}(1-\sigma_{\mathrm{RC}}), c_{\mathrm{m}}(1-\sigma_{\mathrm{MC}}+\sigma_{\mathrm{MB}})\right)\sum_{\tau=1}^{+\infty}\frac{\left(\alpha\left(1-d_{\mathrm{f}}\right)(1-x)\right)^{\tau}}{c_{\mathrm{f}}\left(R_{\bullet}^{\mathbf{f}}-\sigma_{\mathrm{RC}}R_0^{\mathbf{f}}\right)+c_{\mathrm{m}}\left(R_{\bullet}^{\mathbf{m}}-(\sigma_{\mathrm{MC}}-\sigma_{\mathrm{MB}})R_0^{\mathbf{m}}\right)}\begin{pmatrix}R_{\tau}^{\mathbf{f}}\\ R_{\tau}^{\mathbf{m}}\end{pmatrix} \\ &= \widehat{x_{\emptyset}} - x - x\frac{1}{c_{\mathrm{f}}\left(R_{\bullet}^{\mathbf{f}}-\sigma_{\mathrm{RC}}R_0^{\mathbf{f}}\right)+c_{\mathrm{m}}\left(R_{\bullet}^{\mathbf{m}}-(\sigma_{\mathrm{MC}}-\sigma_{\mathrm{MB}})R_0^{\mathbf{m}}\right)}\left(c_{\mathrm{f}}(1-\sigma_{\mathrm{RC}}), c_{\mathrm{m}}(1-\sigma_{\mathrm{MC}}+\sigma_{\mathrm{MB}})\right)\sum_{\tau=1}^{+\infty}\mathbf{V}^{\tau}\begin{pmatrix}R_0^{\mathbf{f}}\\ R_0^{\mathbf{m}}\end{pmatrix} \\ &= \widehat{x_{\emptyset}} - x - x\frac{1}{c_{\mathrm{f}}\left(R_{\bullet}^{\mathbf{f}}-\sigma_{\mathrm{RC}}R_0^{\mathbf{f}}\right)+c_{\mathrm{m}}\left(R_{\bullet}^{\mathbf{m}}-(\sigma_{\mathrm{MC}}-\sigma_{\mathrm{MB}})R_0^{\mathbf{m}}\right)}\left(c_{\mathrm{f}}(1-\sigma_{\mathrm{RC}}), c_{\mathrm{m}}(1-\sigma_{\mathrm{MC}}+\sigma_{\mathrm{MB}})\right)(\mathbf{I}-\mathbf{V})^{-1}\mathbf{V}\begin{pmatrix}R_0^{\mathbf{f}}\\ R_0^{\mathbf{m}}\end{pmatrix} \end{aligned} \tag{S-46}$$

(note that the row vector $(c_{\mathrm{f}}(1-\sigma_{\mathrm{RC}}), c_{\mathrm{m}}(1-\sigma_{\mathrm{MC}}+\sigma_{\mathrm{MB}}))$ is premultiplied). As any nonsingular bidimensional square matrix has analytical formula for its inverse matrix, we can further obtain the analytical expression for $H(x)$ in a element-explicit form, but the resulting equation does not allow for biologically transparent interpretation. Yet, one can recover the equations 10–12 of the main text for $d_{\mathrm{m}} = 0$ and $d_{\mathrm{m}} = 1$ from Eqn (S-46).

No male dispersal

Substituting $d_{\mathrm{m}} = 0$, which leads to $\sigma_{\mathrm{RC}} = \sigma_{\mathrm{MC}} - \sigma_{\mathrm{MB}} = \left(1-d_{\mathrm{f}}\right)^2$, thus yields $c_{\mathrm{f}}(1-\sigma_{\mathrm{RC}}) = c_{\mathrm{f}}(1-\sigma_{\mathrm{RC}})$ and $c_{\mathrm{m}}(1-\sigma_{\mathrm{MC}}+\sigma_{\mathrm{MB}}) = c_{\mathrm{m}}(1-\sigma_{\mathrm{RC}})$; as such $(c_{\mathrm{f}}(1-\sigma_{\mathrm{RC}}), c_{\mathrm{m}}(1-\sigma_{\mathrm{MC}}+\sigma_{\mathrm{MB}})) \propto (c_{\mathrm{f}}, c_{\mathrm{m}})$ holds, and is actually the left eigenvector of $\mathbf{V}$. That is:

$$\mathbf{V} = \alpha\left(1-d_{\mathrm{f}}\right)(1-x)\begin{pmatrix}\frac{1-d_{\mathrm{f}}}{2} & \frac{1-d_{\mathrm{f}}}{2}\\ 1-d_{\mathrm{f}} & 0\end{pmatrix} = \alpha\sigma_{\mathrm{RC}}(1-x)\begin{pmatrix}\frac{1}{2} & \frac{1}{2}\\ 1 & 0\end{pmatrix}, \tag{S-47}$$

with:

$$(c_{\mathrm{f}}(1-\sigma_{\mathrm{RC}}), c_{\mathrm{m}}(1-\sigma_{\mathrm{RC}}))\mathbf{V} = \alpha\sigma_{\mathrm{RC}}(1-x)(c_{\mathrm{f}}(1-\sigma_{\mathrm{RC}}), c_{\mathrm{m}}(1-\sigma_{\mathrm{RC}})) \tag{S-48}$$

(one may remove $1-\sigma_{\mathrm{RC}}$ from this equation). Thus we have:

$$\begin{aligned} (c_{\mathrm{f}}(1-\sigma_{\mathrm{RC}}), c_{\mathrm{m}}(1-\sigma_{\mathrm{RC}}))\sum_{\tau=1}^{+\infty}\mathbf{V}^{\tau}\begin{pmatrix}R_0^{\mathbf{f}}\\ R_0^{\mathbf{m}}\end{pmatrix} &= \sum_{\tau=1}^{+\infty}(c_{\mathrm{f}}(1-\sigma_{\mathrm{RC}}), c_{\mathrm{m}}(1-\sigma_{\mathrm{RC}}))\mathbf{V}^{\tau}\begin{pmatrix}R_0^{\mathbf{f}}\\ R_0^{\mathbf{m}}\end{pmatrix} \\ &= \sum_{\tau=1}^{+\infty}(\alpha\sigma_{\mathrm{RC}}(1-x))^{\tau}(c_{\mathrm{f}}(1-\sigma_{\mathrm{RC}}), c_{\mathrm{m}}(1-\sigma_{\mathrm{RC}}))\begin{pmatrix}R_0^{\mathbf{f}}\\ R_0^{\mathbf{m}}\end{pmatrix} \\ &= \frac{\alpha\sigma_{\mathrm{RC}}(1-x)}{1-\alpha\sigma_{\mathrm{RC}}(1-x)}\left(c_{\mathrm{f}}(1-\sigma_{\mathrm{RC}})R_0^{\mathbf{f}} + c_{\mathrm{m}}(1-\sigma_{\mathrm{RC}})R_0^{\mathbf{m}}\right) \\ &= \frac{\alpha\sigma_{\mathrm{RC}}(1-x)}{1-\alpha\sigma_{\mathrm{RC}}(1-x)}\left(c_{\mathrm{f}}R_0^{\mathbf{f}}(1-\sigma_{\mathrm{RC}}) + c_{\mathrm{m}}R_0^{\mathbf{m}}(1-\sigma_{\mathrm{RC}})\right). \end{aligned} \tag{S-49}$$

Substituting this into Eqn (S-46) supplies:

$$H(x) = \widehat{x_\emptyset} - x - x \cdot \frac{\alpha\sigma_{RC}(1-x)}{1-\alpha\sigma_{RC}(1-x)} \cdot \underbrace{\frac{c_f R_0^{\mathbf{f}}(1-\sigma_{RC}) + c_m R_0^{\mathbf{m}}(1-\sigma_{RC})}{c_f\left(R_\bullet^{\mathbf{f}} - \sigma_{RC} R_0^{\mathbf{f}}\right) + c_m(R_\bullet^{\mathbf{m}} - \sigma_{RC} R_\bullet^{\mathbf{m}})}}_{=\kappa}. \tag{S-50}$$

Using $\kappa = 1/n$ for $d_m = 0$, we recover Eqn (11) of the main text.

Complete male dispersal

Substituting $d_m = 1$ leads to $\sigma_{RC} = \left(1-d_f\right)^2$ and $\sigma_{MC} - \sigma_{MB} = 0$ (hence generating no kin competition in males), which gives:

$$\mathbf{V} = \alpha\sigma_{RC}(1-x)\begin{pmatrix} \frac{1}{2} & 0 \\ 1 & 0 \end{pmatrix}, \tag{S-51}$$

and we immediately get:

$$\mathbf{V}^\tau = \left(\frac{\alpha\sigma_{RC}(1-x)}{2}\right)^\tau \begin{pmatrix} 1 & 0 \\ 2 & 0 \end{pmatrix} \tag{S-52}$$

(for $\tau \geq 1$), which gives:

$$\begin{aligned}(c_f(1-\sigma_{RC}), c_m(1-\sigma_{RC}))\sum_{\tau=1}^{+\infty}\mathbf{V}^\tau\begin{pmatrix} R_0^{\mathbf{f}} \\ R_0^{\mathbf{m}} \end{pmatrix} &= \frac{\frac{\alpha\sigma_{RC}(1-x)}{2}}{1-\frac{\alpha\sigma_{RC}(1-x)}{2}}(c_f(1-\sigma_{RC}), c_m(1-\sigma_{RC}))\begin{pmatrix} 1 & 0 \\ 2 & 0 \end{pmatrix}\begin{pmatrix} R_0^{\mathbf{f}} \\ R_0^{\mathbf{m}} \end{pmatrix} \\ &= \frac{\frac{\alpha\sigma_{RC}(1-x)}{2}}{1-\frac{\alpha\sigma_{RC}(1-x)}{2}}(c_f(1-\sigma_{RC}) + 2c_m(1-\sigma_{RC}))R_0^{\mathbf{f}},\end{aligned} \tag{S-53}$$

where we have used the fact that $R_0^{\mathbf{m}} = 2R_0^{\mathbf{f}}$ when $d_m = 1$ (see Eqn (S-29)). We can therefore see that $(c_f(1-\sigma_{RC}) + 2c_m(1-\sigma_{RC}))R_0^{\mathbf{f}} = c_f(1-\sigma_{RC})R_0^{\mathbf{f}} + c_m(1-\sigma_{RC})R_0^{\mathbf{m}}$, which equals $c_f(1-\sigma_{RC})R_0^{\mathbf{f}} + c_m R_0^{\mathbf{m}}$, and substituting this into Eqn (S-46) yields:

$$H(x) = \widehat{x_\emptyset} - x - x \cdot \frac{\frac{\alpha\sigma_{RC}(1-x)}{2}}{1-\frac{\alpha\sigma_{RC}(1-x)}{2}} \cdot \underbrace{\frac{c_f R_0^{\mathbf{f}}\left(1-\left(1-d_f\right)^2\right) + c_m R_0^{\mathbf{m}}}{c_f\left(R_\bullet^{\mathbf{f}} - \left(1-d_f\right)^2 R_0^{\mathbf{f}}\right) + c_m R_\bullet^{\mathbf{m}}}}_{=\kappa \text{ with } d_m = 1 \text{ inserted}}, \tag{S-54}$$

whereupon we recover Eqn (12) of the main text.

C Extension: multiplivative function of LRE

Model 1: LRE provided from daughters to mothers before dispersal

Here we assume that LRE is generated by a multiplicative functional form, of:

$$\beta_0 = (1-x_0)^{\alpha} K^{\alpha} \beta_0^{\alpha} \tag{S-55}$$

(for $0 \leq \alpha < 1$), which satisfies:

$$\begin{aligned} \beta^{\circ} &= (1-x)^{\frac{\alpha}{1-\alpha}} K^{\frac{\alpha}{1-\alpha}}, \\ \frac{\partial \beta_0}{\partial x_0} &= -\frac{\beta^{\circ}}{1-x} \cdot \frac{\alpha}{1-\alpha} \end{aligned} \tag{S-56}$$

at neutrality. Therefore,

$$g_{\tau=0}(x) = -\Big(c_{\mathrm{f}} Q_0^{\mathbf{f}}(1-\sigma_{\mathrm{RC}}) + c_{\mathrm{m}} Q_0^{\mathbf{m}}(1-\sigma_{\mathrm{MC}}+\sigma_{\mathrm{MB}})\Big)\frac{1}{1-x} \cdot \frac{\alpha}{1-\alpha}. \tag{S-57}$$

Hamilton's rule $H(x)$, which is defined by Eqn (S-26) with $g(x)/Q_{\bullet} = (g_{\emptyset}(x) + g_{\tau=0}(x))/Q_{\bullet}$, is thus given by:

$$H(x) = \widehat{x_{\emptyset}} - x - \kappa \cdot x \cdot \frac{\alpha}{1-\alpha} > 0, \tag{S-58}$$

which determines the cESS as:

$$\widehat{x_{\tau=0}} = \frac{\widehat{x_{\emptyset}}}{1 + \dfrac{\kappa\alpha}{1-\alpha}}. \tag{S-59}$$

This expression immediately tells us that $\alpha \nearrow 1$ leads to $\widehat{x_{\tau=0}} \to 0$.

Model 2: LRE provided from offspring to siblings after dispersal

Similarly, consider:

$$\begin{aligned} \beta_{\tau} &= B\Big(\beta_{\tau+1}, x_{\tau+1}\Big) \\ &= \Big(\Big(1-d_{\mathrm{f}}\Big)(1-x_{\tau+1})\beta_{\tau+1} + d_{\mathrm{f}}(1-x)\overline{\beta}\Big)^{\alpha} K^{\alpha}, \end{aligned} \tag{S-60}$$

which, at equilibrium, should satisfy:

$$\beta^{\circ} = (1-x)^{\frac{\alpha}{1-\alpha}} K^{\frac{\alpha}{1-\alpha}}. \tag{S-61}$$

Partial differentiation gives:

$$\begin{aligned} \frac{\partial \beta_0}{\partial x_1} &= -\alpha \frac{1-d_{\mathrm{f}}}{1-x} \beta^{\circ}, \\ \frac{\partial \beta_{\tau}}{\partial \beta_{\tau+1}} &= \alpha\Big(1-d_{\mathrm{f}}\Big). \end{aligned} \tag{S-62}$$

Hence, with some algebra,

$$g_{\tau\geq 1}(x) = -\frac{\alpha\left(1-d_\mathrm{f}\right)}{1-x}\left(c_\mathrm{f}(1-\sigma_\mathrm{RC}), c_\mathrm{m}(1-\sigma_\mathrm{MC}+\sigma_\mathrm{MB})\right)\left(\mathbf{I}-\alpha\left(1-d_\mathrm{f}\right)\mathbf{P}\right)^{-1}\mathbf{P}\begin{pmatrix} Q_0^\mathbf{f} \\ Q_0^\mathbf{m} \end{pmatrix} \qquad \text{(S-63)}$$

Overall, we can observe that the multiplicative effects of LRE on $\widehat{x}$ are much more pronounced compared to the additive LRE effects (SI Fig 3), which is in part because with this multiplicative formula $x = 1$ gives $\beta = 0$: producing females is a prerequisite for producing offspring.

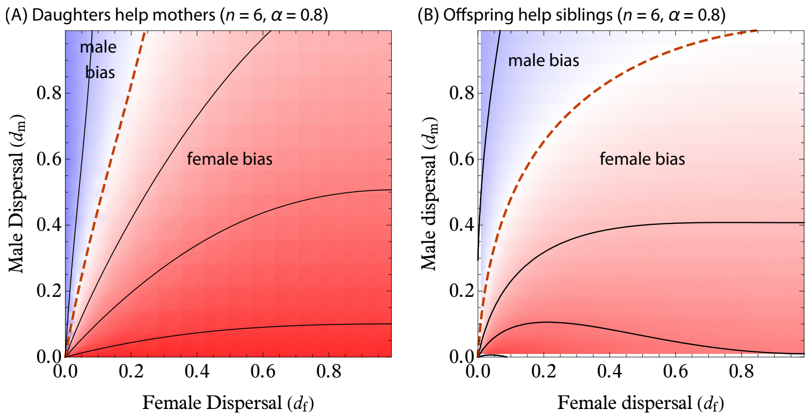

SI Figure 1: cESS plotted against sex-dependent dispersal rates. Orange dotted contours: Fisherian sex ratio ($x = .5$), and the others are ±0.05 steps deviation from Fisherian (0.55, 0.5, 0.45, and 0.40).

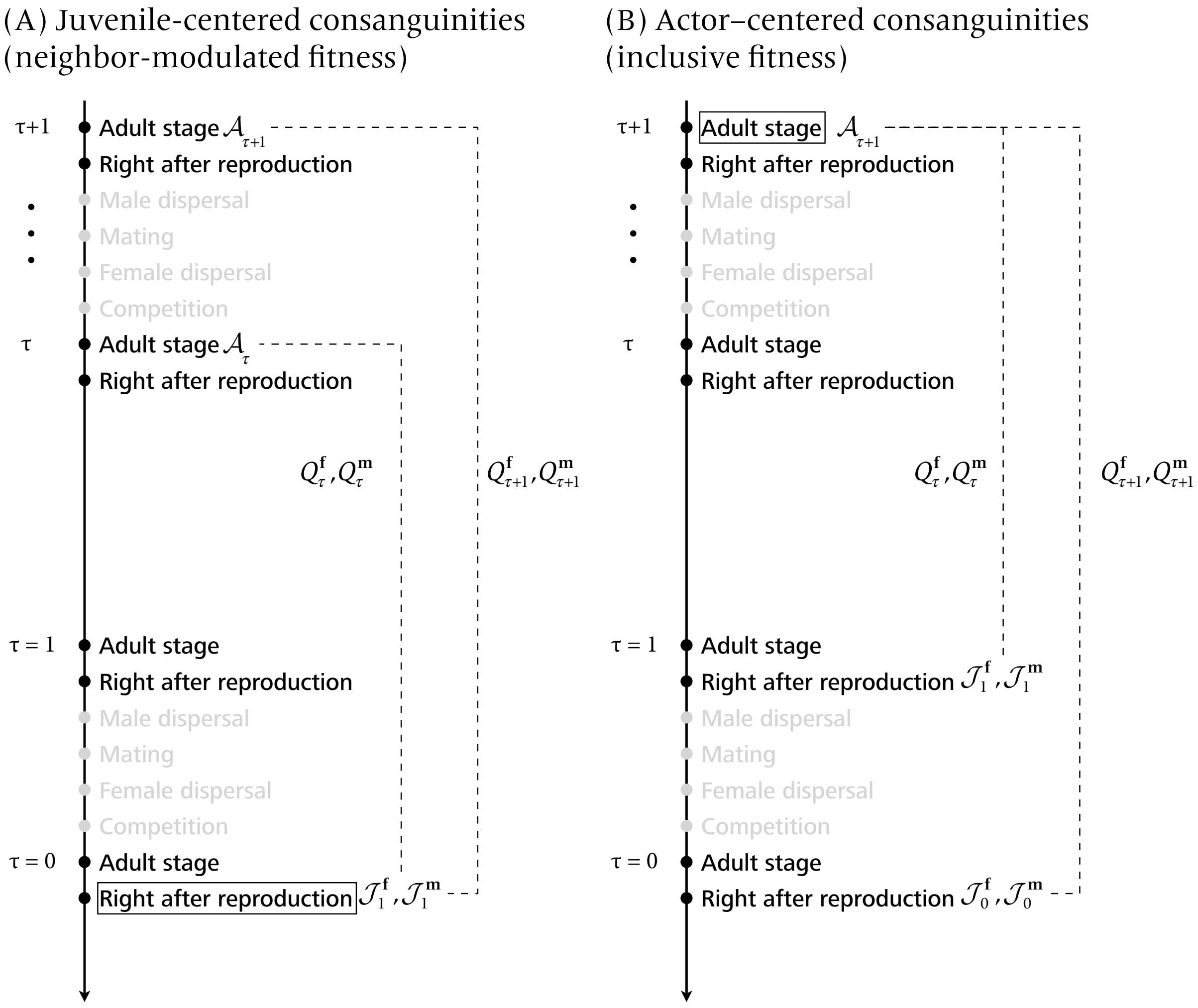

SI Figure 2: Schematic illustration to develop recursive equations for the consanguinity across generations. As we assume that the stochastic process is time-invariant, we can freely shift the time axes τ in the recurrence equations.

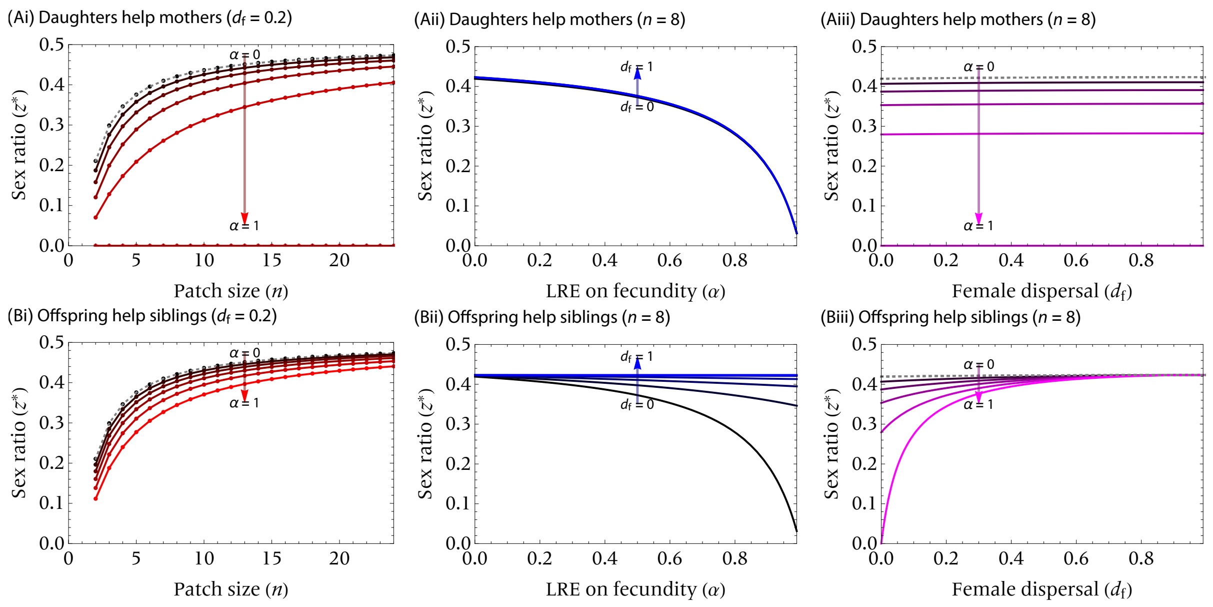

SI Figure 3: cESS when the effect of LRE is of multiplicative function. The male dispersal rate was set $d_m = 0$.

Literatures cited in Appendices

Bulmer, M. G. (1994). *Evolutionary theoretical ecology*. Sinauer Associates, Sunderland, MA.

Caswell, H. (2001). *Matrix population models*. Wiley Online Library.

Frank, S. A. (1998). *Foundations of social evolution*. Princeton University Press.

Gardner, A., Arce, A., & Alpedrinha, J. (2009). Budding dispersal and the sex ratio. *Journal of Evolutionary Biology*, **22**.5, pp. 1036–1045. DOI: 10.1111/j.1420-9101.2009.01719.x.

Johnstone, R. A., Cant, M. A., & Field, J. (2012). Sex-biased dispersal, haplodiploidy and the evolution of helping in social insects. *Proceedings of the Royal Society B: Biological Sciences*, **279**.1729, pp. 787–793. DOI: 10.1098/rspb.2011.1257.

Lehmann, L. (2007). The evolution of trans-generational altruism: kin selection meets niche construction. *Journal of Evolutionary Biology*, **20**.1, pp. 181–189. DOI: 10.1111/j.1420-9101.2006.01202.x.

— (2008). The adaptive dynamics of niche constructing traits in spatially subdivided populations: evolving posthumous extended phenotypes. *Evolution*, **62**.3, pp. 549–566. DOI: 10.1111/j.1558-5646.2007.00291.x.

Michod, R. E. & Hamilton, W. D. (1980). Coefficients of relatedness in sociobiology. *Nature*, **288**.5792, p. 694. DOI: 10.1038/288694a0.

Taylor, P. D. (1988). Inclusive fitness models with two sexes. *Theoretical Population Biology*, **34**.2, pp. 145–168. DOI: 10.1016/0040-5809(88)90039-1.

— (1990). Allele-frequency change in a class-structured population. *The American Naturalist*, **135**, pp. 95–106. DOI: 10.1086/285034.

— (1992). Altruism in viscous populations—an inclusive fitness model. *Evolutionary Ecology*, **6**.4, pp. 352–356. DOI: 10.1007/bf02270971.

Taylor, P. D. & Frank, S. A. (1996). How to make a kin selection model. *Journal of Theoretical Biology*, **180**.1, pp. 27–37. DOI: 10.1006/jtbi.1996.0075.

Taylor, P. D., Wild, G., & Gardner, A. (2007). Direct fitness or inclusive fitness: how shall we model kin selection? *Journal of Evolutionary Biology*, **20**.1, pp. 301–309. DOI: 10.1111/j.1420-9101.2006.01196.x.

Wild, G. & Taylor, P. D. (2004). Kin selection models for the co-evolution of the sex ratio and sex-specific dispersal. *Evolutionary Ecology Research*, **6**.4, pp. 481–502.
